# An in vivo model of glioblastoma radiation resistance identifies long noncoding RNAs and targetable kinases

**DOI:** 10.1172/jci.insight.148717

**Published:** 2022-08-22

**Authors:** Christian T. Stackhouse, Joshua C. Anderson, Zongliang Yue, Thanh Nguyen, Nicholas J. Eustace, Catherine P. Langford, Jelai Wang, James R. Rowland, Chuan Xing, Fady M. Mikhail, Xiangqin Cui, Hasan Alrefai, Ryan E. Bash, Kevin J. Lee, Eddy S. Yang, Anita B. Hjelmeland, C. Ryan Miller, Jake Y. Chen, G. Yancey Gillespie, Christopher D. Willey

**Affiliations:** 1 Department of Neurosurgery,; 2 Department of Radiation Oncology, and; 3 Informatics Institute, Heersink School of Medicine, University of Alabama at Birmingham, Birmingham, Alabama, USA. Birmingham, Alabama, USA.; 4 Department of Physics, The Ohio State University, Columbus, Ohio, USA.; 5 Department of Genetics, Heersink School of Medicine, University of Alabama at Birmingham, Birmingham, Alabama, USA.; 6 Department of Biostatistics and Bioinformatics, Rollins School of Public Health, Emory University, Atlanta, Georgia, USA.; 7 Division of Neuropathology, Department of Pathology, and; 8 Department of Cell, Developmental, and Integrative Biology, Heersink School of Medicine, University of Alabama at Birmingham, Birmingham, Alabama, USA.

**Keywords:** Oncology, Brain cancer, Noncoding RNAs, Radiation therapy

## Abstract

Key molecular regulators of acquired radiation resistance in recurrent glioblastoma (GBM) are largely unknown, with a dearth of accurate preclinical models. To address this, we generated 8 GBM patient-derived xenograft (PDX) models of acquired radiation therapy–selected (RTS) resistance compared with same-patient, treatment-naive (radiation-sensitive, unselected; RTU) PDXs. These likely unique models mimic the longitudinal evolution of patient recurrent tumors following serial radiation therapy. Indeed, while whole-exome sequencing showed retention of major genomic alterations in the RTS lines, we did detect a chromosome 12q14 amplification that was associated with clinical GBM recurrence in 2 RTS models. A potentially novel bioinformatics pipeline was applied to analyze phenotypic, transcriptomic, and kinomic alterations, which identified long noncoding RNAs (lncRNAs) and targetable, PDX-specific kinases. We observed differential transcriptional enrichment of DNA damage repair pathways in our RTS models, which correlated with several lncRNAs. Global kinomic profiling separated RTU and RTS models, but pairwise analyses indicated that there are multiple molecular routes to acquired radiation resistance. RTS model–specific kinases were identified and targeted with clinically relevant small molecule inhibitors. This cohort of in vivo RTS patient-derived models will enable future preclinical therapeutic testing to help overcome the treatment resistance seen in patients with GBM.

## Graphical abstract

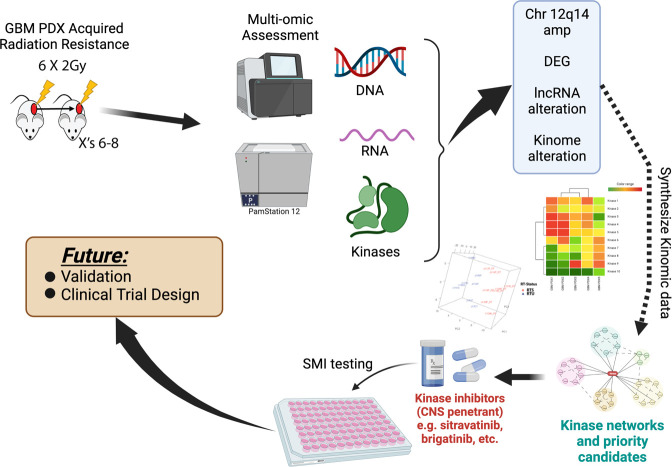

## Introduction

Glioblastoma (GBM) is the most common and devastating form of primary brain cancer. Median survival remains around 15 months despite decades of research (1). Standard adjuvant therapy for GBM includes 6 weeks of fractionated radiotherapy (RT) (typical total dose around 60 Gy) with concomitant systemic therapy using the alkylating agent temozolomide (TMZ) (75 mg/m^2^ daily), followed by 6–12 months of adjuvant TMZ (150–200 mg/m^2^ for 5 days every 28 days) (2). While O^6^-methylguanine-DNA methyltransferase promoter methylation is a known predictive marker for GBM response to TMZ (3), there are no validated predictive or prognostic molecular indicators for RT response in GBM (4). GBM almost invariably recurs, and recurrent tumors frequently acquire resistance to conventional therapies. In recent years, several potential radiation resistance mechanisms have been postulated, including transcriptional reprogramming (5), epigenetics (6), cell state plasticity (7), irradiation changes to normal brain tissue (8, 9), metabolic alterations (10), and epithelial-mesenchymal transition (11), to name a few. Still, there is a dearth of accurate preclinical models, especially of recurrent, therapy-resistant tumors, which are in most cases responsible for patient mortality (12).

Here we have generated 8 pairs of GBM patient-derived xenografts (PDXs) in which the radiation-sensitive, unselected (RTU) tumors were irradiated for in vivo serial selection to create recurrent, radiation therapy–selected (RTS) variant PDXs. This models a common therapeutic strategy whose outcomes unfortunately lead to acquired therapy resistance in patients (13). These patient-matched, paired PDXs allow the comparison of primary and recurrent tumors to better understand the trajectories of molecular mechanisms in tumor recurrence.

Two recent longitudinal studies in glioma have reported that selective pressures and driver mutations tend to occur early in glioma development, with little change at the genomic level following recurrence (14, 15). This may suggest that phenotypic changes such as acquired therapy resistance are not driven at the genomic level but instead at the epigenetic, transcriptomic, and posttranslational levels. To this end, we have conducted whole-exome sequencing (WES) as well as deep RNA sequencing (RNA-Seq) of total RNA in order to study transcripts including long noncoding RNAs (lncRNAs). LncRNAs can act as molecular sponges for microRNAs or transcription factors, scaffolds for enhancer or repressive complexes, guides for chromatin remodeling enzymes, or as signals for gene activation (16, 17). The Pan-Cancer Analysis of Whole Genomes consortium has recently identified 122 lncRNAs with causal roles in cancer tumorigenesis, a number of which overlap with our results (18).

In order to identify lncRNAs and their potential targets, we have devised an in silico approach using quasi-mapping of long, paired-end reads (19) combined with nucleic acid binding prediction software using thermodynamics-based algorithms to detect RNA:RNA duplex or RNA:DNA triplex formation (20, 21) (see Methods section). We also combine traditional differential gene expression analysis (DE) with differential gene correlation analysis (DGCA) (22), machine learning (23, 24), and semantic network construction (25, 26) to further elucidate transcriptional mechanisms of therapy resistance. These approaches have revealed differential regulation of DNA damage response (DDR) pathways as well as stemness, cell cycle, and chromatin remodeling signatures. We have noted differential enrichment of canonical DDR pathways at the transcriptomic level in vivo after selection. A number of lncRNAs are associated with the differential expression of transcripts in these pathways.

We evaluated our RTS models by integrating global kinase (kinomic) activity and transcriptomic data to identify radiation-induced changes in baseline signaling and expression. Kinase signaling is highly therapeutically targetable in neoplasms (27), and radiation therapy has been demonstrated to induce malignant phenotypes through modulation of Src and tropomyosin receptor kinase (Trk) family kinase signaling among others (28, 29). Our results showed that radiation therapy selection induced targetable kinase activity alterations. Clinically used small molecule inhibitors (SMIs) sitravatinib and brigatinib (30, 31), with known brain penetrance, were effective in RTS GBM models. The application of our integrated informatics approach using these clinically relevant models provides a method for selecting preclinical therapies for intractable, therapy-resistant tumors.

## Results

### Serial in vivo radiation of GBM PDXs generates RTS derivatives.

**Figure 1:**
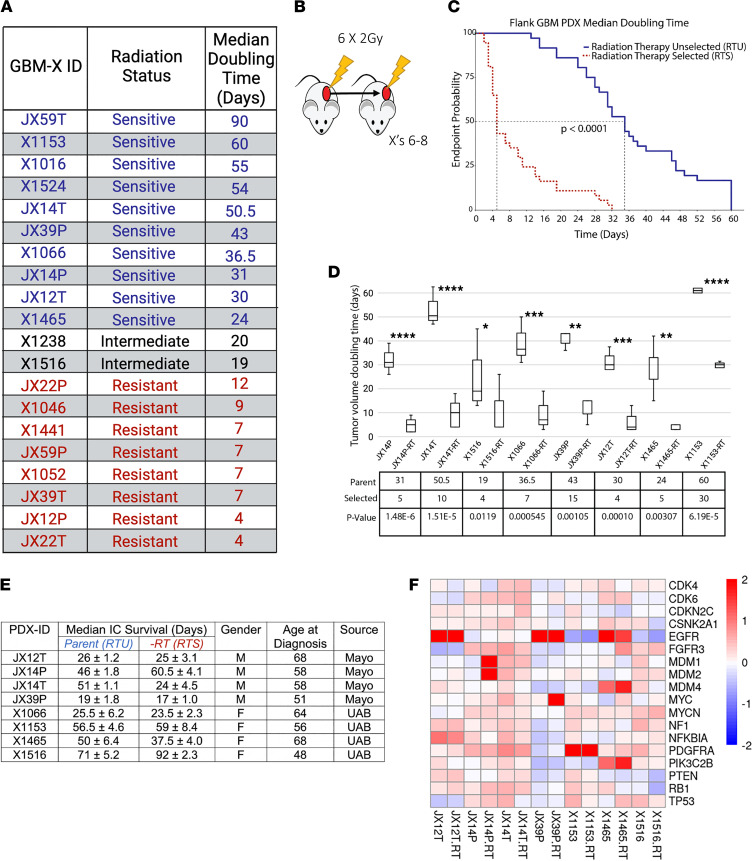
GBM PDX RT-selected models exhibit differences in survival and molecular diversity. (**A**) Initial RT sensitivity status and median doubling times for tumors after RT. (**B**) Serial in vivo RT selection methodology. (**C**) RTS (red dashed line) and RTU (blue solid line) combined endpoint probabilities following initiation of radiation treatment. *P* value calculated from log-rank test. (**D**) Changes in median doubling time pre- and post-RT selection. The box plots depict the minimum and maximum values (whiskers), the upper and lower quartiles, and the median. The length of the box represents the interquartile range. *P < 0.05, **P < 0.01, ***P < 0.001, and ****P < 0.0001. *P* values calculated by 2-sided *t* test. (**E**) Basic characteristics of PDXs used for RTS with patient sex (M, male; F, female), patient age, and original source. UAB, University of Alabama at Birmingham. (**F**) Log_2_ fold change copy number calls from patternCNV for GBM hallmark genes. Median survival intracranial (*n* = 6). Median doubling time (*n* = 5) per pair (*n* = 80 total).

A cohort of 20 heterotopic (subcutaneous) GBM PDXs were screened for sensitivity to radiation. Tumor growth was measured externally using calipers, and radiation response was defined as “resistant” (*n* = 8) if the median-doubling time of the initial tumor volume was significantly less than 20 days, “sensitive” (*n* = 10) for doubling times greater than 20 days, or “intermediate” (*n* = 2) if not significantly different from 20 days (Figure 1A). Of these, existing Affymetrix transcriptomic microarray data were available for 13 PDX lines (*n* = 7 sensitive, *n* = 6 resistant). We found that 18 genes were significantly upregulated, and 8 genes were significantly downregulated in the inherent resistant group (Supplemental Figure 1A; supplemental material available online with this article; https://doi.org/10.1172/jci.insight.148717DS1). Overrepresentation analysis of these 26 genes revealed no significant enrichment at an FDR of less than 0.05; therefore, we took the top 10 results (Supplemental Figure 1B). CXCR3 receptor binding was the top result driven by the significant downregulation of ligands CXCL10 and CXCL11 in inherently resistant PDXs. Protein binding was the most enriched molecular function Gene Ontology term followed by ion binding (Supplemental Figure 1C). The top enriched gene set from gene set enrichment analysis (GSEA) using the Molecular Signatures Database (MSigDB) H and C6 sets was hallmark coagulation, but the BCAT.100_UP.V1_DN set was also significantly enriched (Supplemental Figure 1D). This was primarily driven by high snail family transcriptional repressor 2 expression in resistant PDXs (one of the top overexpressed genes in DE). Enrichment of this set indicates enhanced β-catenin expression/activity in inherently resistant PDXs. While inherent radiation resistance has importance, acquired (or adaptive) radiation resistance is the more pressing clinical problem for GBM. Therefore, we sought to develop adaptive radiation-resistant models. Eleven PDXs (RTU) underwent serial in vivo selection (6–8 serial passages) against radiation therapy (Figure 1B). Three of these lines were consistently “cured” by radiation therapy and were thus unable to complete the radiation selection process. However, Figure 1C shows the difference in doubling time as combined endpoint probabilities of the remaining sensitive, RTU lines (solid blue line) and their paired resistant (RTS) lines (dashed red line). The median endpoint probability for the doubling time of the combined RTU lines was reduced by 85% compared with the median of the RTS lines from 35 to 5 days (*P* < 0.001). Eight PDXs were successfully selected for resistance to radiation with parent and selected tumor doubling times as indicated (Figure 1D). Basic clinical demographic information for these lines (Figure 1E) and a GBM hallmark driver gene panel (Figure 1F) are shown. Of note, CDKN2A/B were deeply deleted in most samples, and JX39P and JX39P-RTS harbor the EGFR_VIII_ variant.

### Chromosome 12q14 amplification identified in some RTS PDXs.

**Figure 2:**
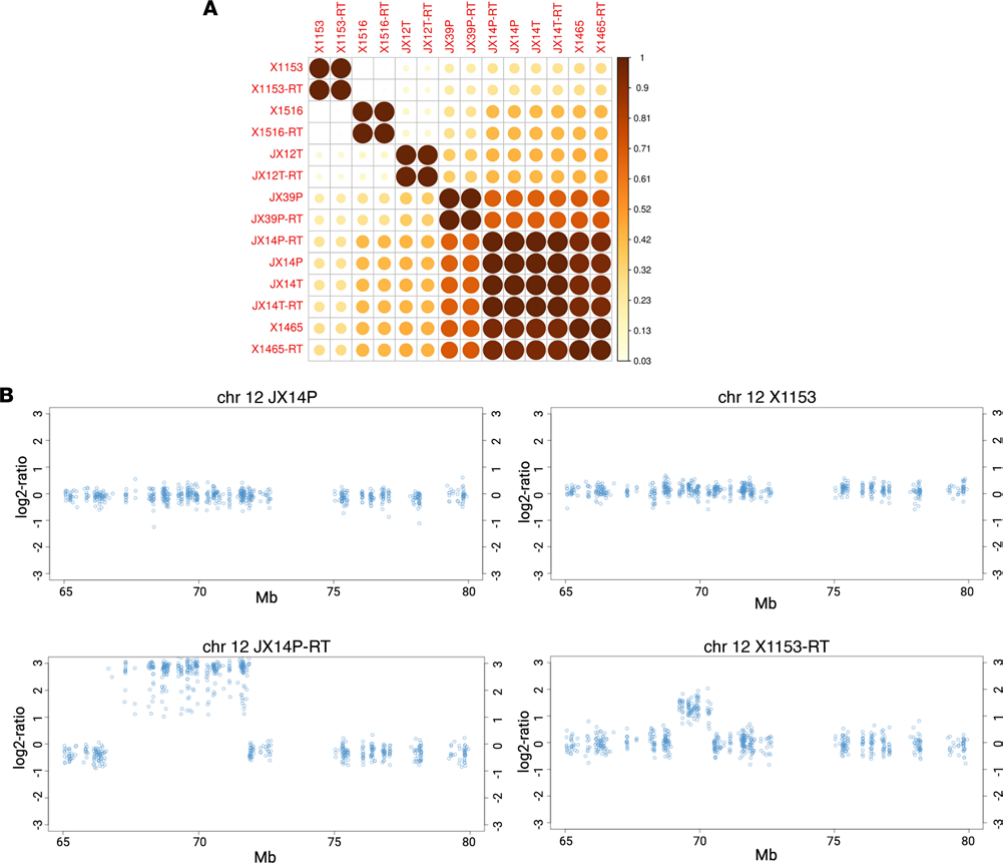
Identification of chromosome 12q14 amplification in select GBM PDX-RT models. (**A**) Multidimensional scaling (MDS) of SNP correlations from whole-exome sequencing (WES) data. A distance matrix was calculated between each sample, and a corrplot was made with 1 – (Euclidean distance) of the MDS plot. (**B**) PatternCNV output zoomed-in view for amplification locus in chromosome 12q for JX14P and JX14P-RT (left panel) and X1153 and X1153-RT (right panel).

SNP analyses indicated that RTS PDX lines correlated highly with their parent RTU lines (Figure 2A). Like the GBM hallmark driver gene panel data, these results suggest that RTS PDX lines mirror clinical data sets (14) by generally maintaining their classic genomic alterations. However, 2 of the 8 RTS lines, X1153-RTS and JX14P-RTS, were found to have a copy number amplification on chromosome 12q14 (Figure 2B), a locus previously identified in patients with recurrent GBM. While 12q amplification could suggest a possible mechanism for the acquired radiation resistance in the RTS lines, we anticipated that transcriptome and kinome assessment may be more revealing.

### Transcriptomic analysis identifies differentially regulated lncRNAs that may have functional roles.

**Figure 3:**
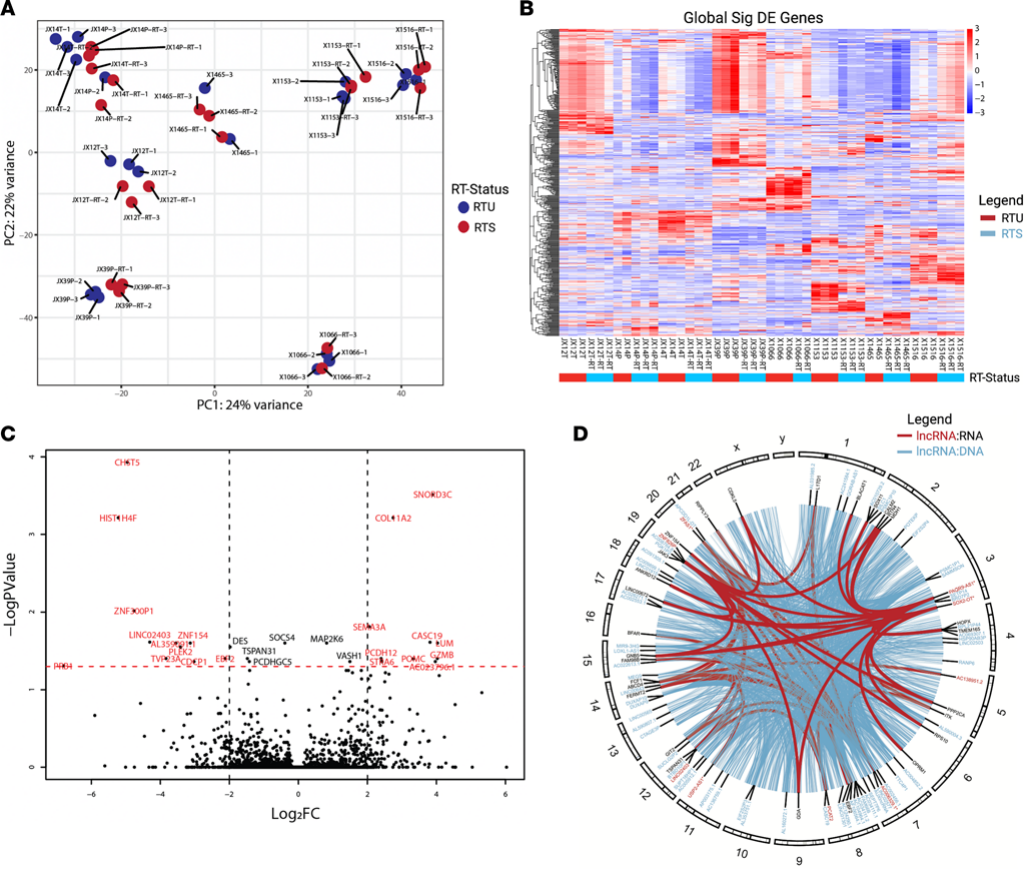
GBM PDXs grossly separate according to patient tumor of origin, with a small number of significantly differentially expressed genes between RTU and RTS. (**A**) PCA of global transcriptomic differences. (**B**) Heatmap of globally DEGs when modeled as ~ PDX_ID + RTS. (**C**) Volcano plot of globally DEGs when modeled by ~RTS alone (red text meets cutoffs of log_2_ fold change > 2 or < –2, adjusted *P* < 0.05). (**D**) Circos plot of differentially regulated lncRNAs (red) with their direct DNA binding targets (black) and differentially regulated lncRNAs (blue) with their DNA binding sites.

Intracranial orthotopic xenografts were established, and tumors were harvested in biological triplicate for omics analysis (32). Each orthotopic tumor was divided in half for matched kinomic and transcriptomic evaluation. Globally, at the transcriptome level, PDX pairs primarily separated based on patient of origin, with some smaller separation based on radiation sensitivity on principal component analysis (PCA, Figure 3A). Global DE including patient tumor of origin and selection status (~PDX_ID + RTS) revealed 482 significantly differentially expressed genes (DEGs) (Figure 3B), including 69 lncRNAs and 24 pseudogenes. Global DE between the combined RTU versus RTS (~RTS), without patient tumor of origin as a covariate, revealed 27 significantly DEGs (Figure 3C), including 5 lncRNAs and 1 pseudogene. One of these lncRNAs, cancer susceptibility 19 (CASC19/PCAT2) (ensembl ENSG00000254166.3), was globally upregulated in selected PDXs (adjusted *P* = 0.02) (Supplemental Figure 2A), particularly in JX39P, and had predicted DNA binding at several genomic sites (method described in Supplemental Figure 2B), including sites proximal to mitogen-activated protein kinase 6 (MAP2K6); another globally DEG, and sites on the FES proto-oncogene. In the JX39P pair, CASC19 expression is positively correlated with expression of MAP2K6 and FES (Supplemental Figure 2C). Additional differential expression analytic approaches were performed, including 2 machine learning methods (FastEMC and WGCNA) and DGCA that both identified several lncRNAs and pseudogenes being differentially expressed in RTS models that were used for lncRNA correlations (Supplemental Figure 3).

To elucidate potential regulatory roles of the differentially expressed lncRNAs, a pipeline incorporated RNA:RNA (ASSA) and RNA:DNA (Triplexator) binding prediction software (20, 21) with bedtools to identify proximal genes to lncRNA:DNA binding sites (Supplemental Figure 2B). Presumptive cis-regulatory targets for the significantly altered lncRNAs were identified as genes that were common to both the lncRNA:DNA proximal gene list and the pairwise DEG list. Nine lncRNAs were predicted to bind directly to a combined 28 differentially regulated genes identified in upstream analyses (Figure 3D). DNA binding potential was discovered for 45 lncRNAs at several thousand sites across regulatory regions of the human genome (Figure 3D). Some lncRNAs (CASC19/PCAT2, SOX2-OT, ZFAS1, PAQR9-AS1, and USP2-AS1) possessed both RNA:RNA and RNA:DNA binding potential. Searching a 20 kb window upstream and downstream from these predicted DNA binding sites, we discovered 4268 proximal genes (Figure 3D). Correlative analysis revealed both strong positive and negative correlations between the expression of lncRNAs and proximal genes (Supplemental Figure 3 and Supplemental Data 1). The highest correlated genes were associated with several cancers or cancer-related pathways, including GBM, astrocytoma, neuroblastoma, leukemia, lymphoma, hepatocellular carcinoma, gastric cancer, stemness, angiogenesis, invasion, proliferation, and DDR (Supplemental Data 1). Specific pairwise correlations are detailed in Supplemental Figure 4, and gene interaction networks (WIPER) are shown in Supplemental Data 2.

### Acquired radiation-resistant tumors display distinct DDR pathway alterations.

**Figure 4:**
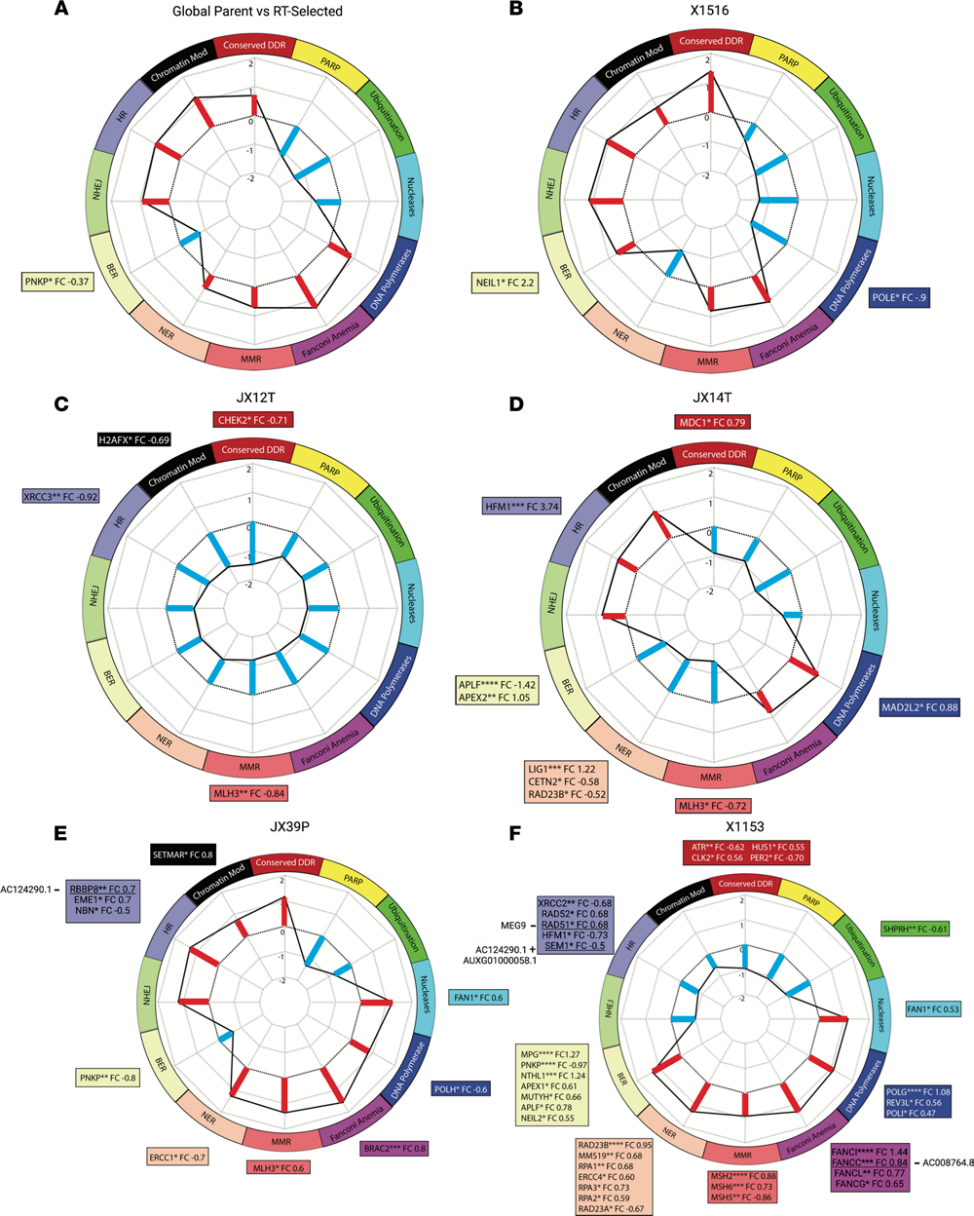
Differential enrichment of DDR pathways and response to DNA damage in PDX pairs. Enrichment of DDR pathways in RTS globally (**A**), in X1516 (**B**), in JX12T (**C**), in JX14T (**D**), in JX39P (**E**), and in X1153 (**F**). The outside edge of the radar plot is labeled and color-coded by DDR pathway. Lines and bars within the plot indicate the normalized enrichment score (–2 to 2) for each DDR signature. Red bars indicate enrichment in the RTS PDX, and blue bars indicate enrichment in the RTU PDX. Significantly DEGs within each pathway along with their significance level and log_2_ fold change are listed in the boxes color-coded to match the DDR pathways in the radar plot. *P* values for differential expression are from Fisher’s exact test. Underlined genes are correlated with the expression of lncRNAs, which are labeled next to the DDR gene. (+) indicates a positive correlation with expression and (–) indicates a negative correlation. *P < 0.05, **P < 0.01, ***P < 0.001, and ****P < 0.0001. HR, homologous recombination; NHEJ, nonhomologous end joining; BER, base excision repair; NER, nucleotide excision repair; MMR, mismatch repair; Mod, modification.

One of the hallmarks of radiation resistance is increased or altered DDR activity. Figure 4, A–F, show the normalized enrichment scores of RTS versus RTU PDXs for 12 DDR transcriptional signatures. It was not possible to generate normalized enrichment scores for JX12P, X1465, or X1066 due to the imbalanced number of replicates. Globally, as well as in the JX39P pair, Fanconi anemia (FA), MMR, NER, NHEJ, HR, and chromatin modification signatures were enriched in RTS PDXs (Figure 4, A and E). X1516 also showed similar DDR enrichment to global, except that BER was enriched in RTS PDXs with NER conversely decreased (Figure 4B). The JX14T pair had similar enrichment to global and JX39P, except that NER and MMR were significantly decreased in the RTS lines (Figure 4D). In the X1153 pair, BER, NER, MMR, and FA were enriched in RTS PDXs, while HR, NHEJ, and chromatin modification were decreased in RTS PDXs (Figure 4F). The JX12T pair was the most divergent from the other RTS PDXs, with all 12 DDR signature normalized enrichment scores decreased for RTS over RTU PDXs (Figure 4C).

Several lncRNAs showed strong associations with significantly differentially expressed DDR genes. In the X1153 pair, AC008764.8 showed a negative correlation with FA-related gene FANCC (Figure 4F and Supplemental Figure 3). Also, AC124290.1 and AUXG01000058.1 showed strong positive correlations with the HR-related gene SEM1 (Figure 4F and Supplemental Figure 3). SOX2-OT was positively correlated with NHEJ-related XRCC4 and to a lesser extent with RAD9A (Supplemental Figure 3). In the JX39P pair, AC002456.1 was negatively correlated with HR-related RBBP8 (Figure 4E) and positively correlated with NER-related MNAT1 and NHEJ-related XRCC5 (Supplemental Figure 3).

### Kinome profiling identifies PDX-specific actionable targets in RTS PDXs.

**Figure 5:**
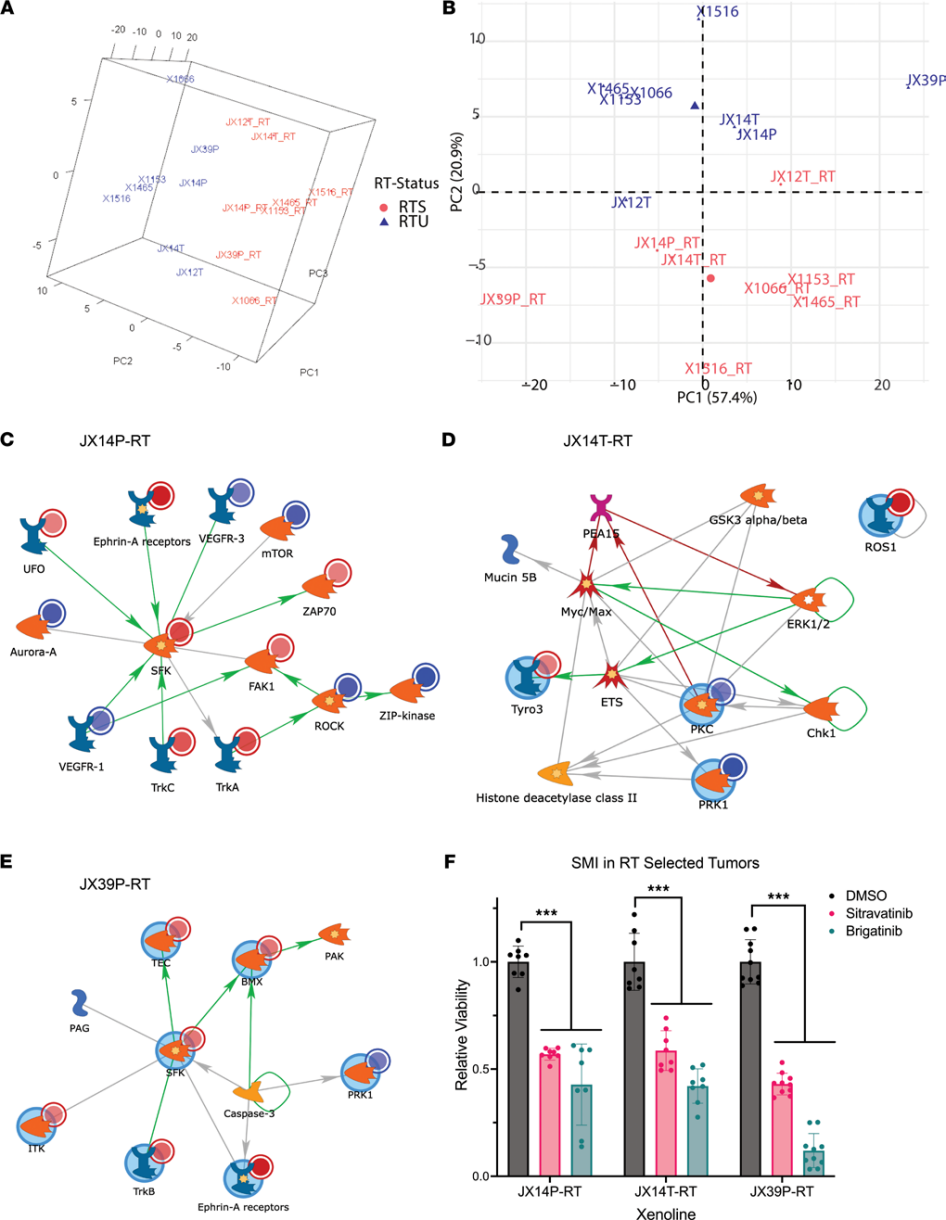
Kinase signaling alterations in RTS targeted with SMIs. PCA demonstrates separation of kinomic signal signatures across 3 components (**A**), colored by RTS (red) and RTU (blue) with the first 2 components plotted (**B**). Kinases altered in RTS compared with RTU for JX14P (**C**), JX14T (**D**), and JX39P (**E**) were modeled with GeneGo MetaCore direct-interaction or auto-expand < 20 node networks. Uploaded kinases are indicated with circles as RTS increased (red) or decreased (blue). Lines between nodes indicate interactions with color indicating type (green; positive, red; negative; gray; other). PDX tumor cells were grown as neurospheres, and viability was measured after 7-day treatment (500 nM sitravatinib or brigatinib) with CellTiter-Glo (CTG) (**F**) and displayed as percentage of vehicle control with bars for SEM, with ***P < 0.0001 calculated using 2-way ANOVA.

Global kinomic profiling of RTU and RTS pairs revealed common and PDX-specific alterations in kinase signaling. As opposed to global transcriptomic analysis, the kinomic profiles separated PDXs primarily by resistance status (RTU or RTS) (Figure 5, A and B). Globally, kinase signaling was decreased in RTS compared with RTU, when the samples were analyzed together. However, this appeared to be driven by a small subset of samples, such as X1066 and X1516, that had relatively large kinase activity decreases in RTS compared with RTU (Supplemental Data 3). Across PDX pairs there was large variation in altered kinase signaling, with some RTS having globally decreased activity in Src family kinase and growth factor pathways (X1066, Supplemental Data 3), as well as PKC-centric decreases (X1516).

Next, upstream kinases alone, and integrated with differentially regulated lncRNA:DNA binding proximal genes, were analyzed at the pathway level (Supplemental Data 4). Src family kinase activity was upregulated in RTS in the integrated analysis of JX14P and JX39P while being decreased in X1153 and X1465 RTS. Ephrin receptor activity was increased in RTS variants of JX14P and X1465. We observed increased activity of EGFR and VEGFR signaling in X1153. As we are interested in evaluating pharmaceutical targets, we focus here on those actionable kinases that exhibited increased activity in the RTS PDXs.

To test the druggable potential of the kinases increased in Figure 5, C–E, and Supplemental Data 4, in vitro SMI screening was conducted using sitravatinib and brigatinib in PDX-derived neurosphere cultures. Sitravatinib targets TRKA/B (JX14P-RTS, JX39P-RTS), ROS1 (JX14T-RTS), Src (JX14P-RTS, JX39P-RTS), as well as multiple Ephrin isoforms. Brigatinib targets ROS1 (JX14T-RTS) and the canonical GBM target EGFR (JX39P-RTS) (30, 31). We observed that JX14T-RTS, JX39P-RTS, and JX14P-RTS were significantly sensitive to both sitravatinib and brigatinib (Figure 5F).

**Figure 6:**
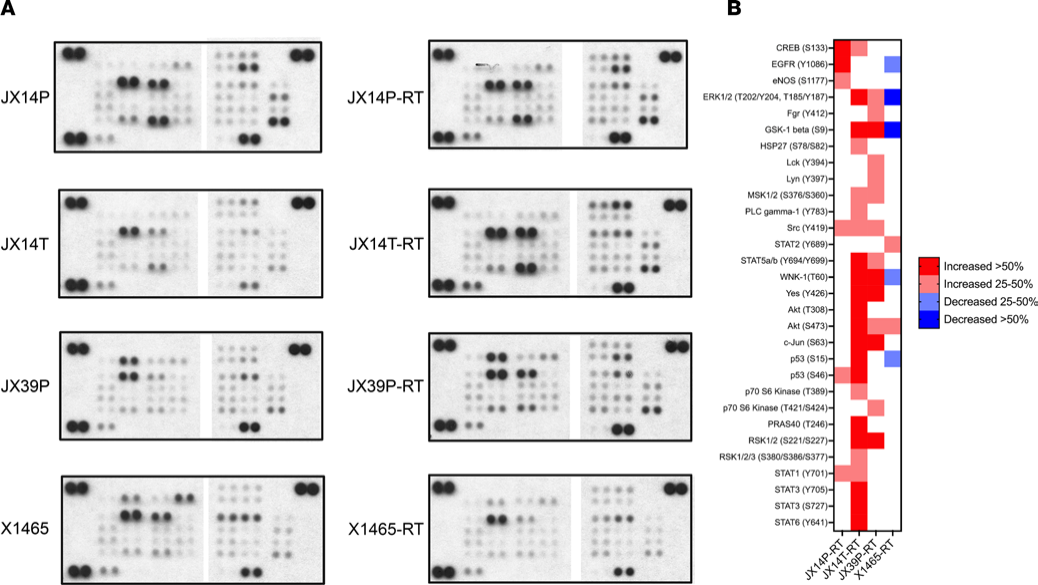
Phospho-kinase Western blot array shows differential phosphorylated proteins in GBM PDX-RTS pairs. (**A**) R&D Systems Proteome Profiler Human Phospho-Kinase Arrays for RTU and RTS pairs for JX14P, JX14T, JX39P, and X1465 shown with equal protein loading. (**B**) Heatmap showing relative change in phospho-antibody spot intensity with at least 25% difference as compared with parent tumor signal.

Confirmatory Western blot array analyses were performed using the R&D Systems Proteome Profiler Human Phospho-Kinase Arrays on paired RTU and RTS from JX14P, JX14T, JX39P, and X1465. As shown in Figure 6, A and B, the RTS lines for JX14P, JX14T, and JX39P exhibited increased phosphorylation of multiple kinases and/or kinase targets, including Src family kinases (SFKs) (Figure 5). However, X1465-RTS showed phosphorylation decreases or no appreciable changes compared to its RTU line except for STAT2 (Y689) and AKT (S473), which were modestly elevated. These results supported the PamStation kinomic profiling of X1465-RTS, which showed decreased SFK activity relative to its RTU parent (Supplemental Data 4). Additionally, an upstream kinase comparison was performed to integrate the PamStation kinomic profiles and the R&D Systems based on the Phosphonet database (see Supplemental Figure 5). Top increased kinases, validated from the previous kinomic screen, included increased Hck, Yes1, and Src (JX14P-RTS); increased Lyn and Src (JX39P-RTS); and increased JNK2 (X1465-RTS). Across 3 of the 4 RTS lines, there were increases in activity of Src family, PIM family, MSK1, PYK2, and JNK kinases. Additionally, BRK was activated as the top kinase in JX39P-RTS and scored high in JX14P-RTS and JX14T-RTS as compared with parental RTU lines (Supplemental Figure 5).

## Discussion

We generated, characterized, and validated several GBM PDX models of radiation resistance (RTS) from their radiation-sensitive parental (RTU) PDXs and identified levels of transcriptomic and phenotypic heterogeneity and mechanisms of acquired resistance. We qualitatively observed a general increase in the invasive potential of orthotopic RTS PDXs when dissecting intracranial tumors and quantitatively showed that RTS tumors were more aggressive with substantial reduction in orthotopic PDX overall survival in vivo. We performed genomic, transcriptomic, and functional proteomic (kinomic) testing on these models with results supporting our contention that these models are clinically relevant. Indeed, we found that mutational drivers were generally retained upon radiation selection, a finding previously observed in GBM patients with longitudinal molecular testing (14, 15). When we did detect new amplifications, they tended to be supported by the clinical literature (33), such as the chromosome 12q14 amplification seen in 2 of the 8 RTS pairs. Transcriptomic analyses demonstrated the tumor models tended to group based on the tumor of origin rather than RTS status, suggesting that acquired radiation resistance may be context dependent. Within individual GBM PDX pairs, however, our transcriptomic analyses suggested several potential pathways to acquired radiation resistance. Additionally, there were RTS-mediated differences in the enrichment of numerous GBM-related gene signatures, including stemness, cell cycle, chromatin remodeling, IFN/STAT1 signaling, and molecular subtypes.

We observed a contrast in the type of changes from RTU to RTS between global transcriptomic and global kinomic profiling. At the kinome level, samples separated clearly based on radiation selection status and not by patient tumor of origin. This speaks to the downstream, function-level effects of radiation selection with relatively fewer changes at the transcriptional level. Gross transcription appears to be driven by the inherent genetic background of the original patient tumor, while the acquired resistance phenotype is functionally distinct at the kinome level. This could suggest that small differences at the transcriptomic level, possibly in lncRNA expression, could have large downstream functional impacts. This is consistent with our knowledge that lncRNAs can have diverse, often concurrent, molecular roles despite their relatively low abundance (17).

We detected both common and PDX-specific differential enrichment of upstream kinases between RTU and RTS PDXs. In X1066 and X1516, kinase activity was mostly repressed in RTS PDXs, potentially indicating a more homeostatic condition, characteristic of senescent or stem-like cells. In some cases, activities of SFKs (JX14P/T, JX39P) and EGFR (X1465, JX39P, JX12T) are increased in RTS PDXs. Brigatinib, an anaplastic lymphoma kinase and EGFR inhibitor, was effective at killing radiation-resistant cells in 3 selected RTS PDX lines (Figure 5F). Sitravatinib, targeting Trk, Src, and Ephrins among others, was also efficacious in these 3 PDX lines (Figure 5F). Of note, brigatinib has demonstrated clinical intracranial efficacy for lung cancer brain metastases, suggesting potential application in recurrent GBM (34). Sitravatinib has been identified as a potential therapy for overcoming immune checkpoint blockade resistance (35). As targeting the immune checkpoint blockade has had limited clinical benefit in GBM, it is possible that combining it with drugs such as sitravatinib would be more beneficial. Src and Trk family kinases are induced by radiation treatment in breast cancer cells and in human umbilical vein endothelial cells (28, 29), while Src has long been considered a therapeutic target in GBM (36). Recently published work has shown that in vitro radiation selection of human and mouse-derived glioma stem cells can induce the insulin like growth factor 1 receptor–mediated (IGF1R-mediated) resistance pathway via N-cadherin upregulation. IGF1R inhibition could reverse this radiation resistance, which was validated in 2 of our RTS lines, JX14P-RTS and JX39P-RTS (11). Together, these findings suggest that resistance models can be used to identify druggable targets for recurrent, therapy-selected tumors.

RTS PDX models displayed increased in vivo resistance to RT, though there is diversity in the enrichment of DDR signatures (Figure 4), indicating potentially different routes to acquired radiation resistance. Given the high degrees of intra- and intertumor heterogeneity observed in GBM, it is expected that each individual tumor could adapt differently under therapeutic pressures. Our cohort of selected tumor pairs provides a wide range of molecularly diverse phenotypes for interrogation. Almost universally (excepting JX12T), FA-related DDR was enriched in RTS PDXs. The FA core complex of 8 proteins is located in the nucleus and responsible for surveillance for DNA double-stranded breaks (37). This complex ubiquitinates FANCD2, which is also phosphorylated by ATM, prompting its interaction with the BRCA1/2 complex that also contains RAD51 (38, 39). In this way, FA genes promote DDR primarily through the high-fidelity HR pathway (40). The enrichment of the FA-related gene signature may therefore indicate a preferential reliance on this surveillance mechanism in acquired radiation resistance. HR and NHEJ were also frequently enriched in RTS PDXs, the exceptions being JX12T and X1153. X1153-RTS PDX appears to rely more on BER, NER, and MMR mechanisms. Interestingly, GSEA using our DDR gene sets revealed an enrichment of 10 out of 12 signatures in inherently resistant tumors, but only NER passed the FDR significance threshold of <0.25. On the whole, differential basal expression, perhaps due to distinct genetic/epigenetic backgrounds, described most of the variance in the model. Radiation resistance status captured a small amount of the variance, supporting our thinking that radiation resistance is mediated at a posttranscriptomic/epigenetic level.

Many DDR genes are located proximal to significantly differentially regulated lncRNAs with DNA binding sites in the regulatory genome. Some of these lncRNA:DDR gene associations show positive and/or negative correlations in expression, especially in FA and HR (Figure 4, E and F, and Supplemental Figure 3). Our data suggest a relationship between lncRNAs and DDR pathway modulation. It is possible that this is accomplished through epigenetic regulation caused by the direct binding of lncRNAs to regulatory regions of DNA proximal to DDR gene loci.

Our in silico pipeline identified 184 lncRNAs differentially regulated between radiation-sensitive RTU and RTS PDXs. Some, SOX2-OT, ZFAS1, SAMMSON, CASC19/PCAT2, and PVT1, have already been associated with various cancers, including gliomas (18, 41, 42). The majority of the lncRNAs identified in this study represent potentially novel transcripts with limited to no information available about their associations or mechanisms. We evaluated the RNA binding potential of these lncRNA transcripts. Nine lncRNAs had predicted RNA binding capacities with such targets as ZNF154, a putative tumor suppressor in nasopharyngeal carcinoma and prostate cancer (43, 44); JAK3, a tyrosine kinase implicated in leukemias and lymphomas (45, 46); and SOX11, which may act as a tumor promoter or suppressor in various cancers, including glioma (47). Forty-five lncRNAs are predicted to bind DNA in regulatory regions of the human genome and have strong correlations with DEGs proximal to those binding sites. We have observed that associations with lncRNA and their potential targets can be either negative or positive, similar to the DDR relationship.

The lncRNA AF106564.1 demonstrated positive correlations with the FIP1L1-PDGFRA fusion transcript and with neurotrophic receptor tyrosine kinase 3 (NTRK3) (Supplemental Data 1). Fluorescent in situ hybridization using the Vysis LSI 4q12 Tri-Color rearrangement probe was not able to confirm the presence of a canonical deletion in the 4q12 region that leads to the FIP1L1-PDGFRA fusion product in heterotopic PDX. This is a limitation as the transcript level detection was performed with orthotopic and not with heterotopic PDXs, highlighting the differences in molecular signaling dependent on locations of tumor implantation. Amplification of this region may lead to poorer outcomes as PDGFRA is a putative oncogene in glioma and FIP1L1 is constitutively expressed in oligodendrocyte precursor cells (48). The association of AF106564.1 with NTRK3 (ensembl ENSG00000140538) is also intriguing as NTRK genes code for tyrosine kinase receptors for growth factors in the CNS and activating fusions such as ETV6-NTRK3 or BTBD1-NTRK3 have oncogenic potential in some subsets of high-grade glioma (49). Coexpression of these pairs of genes is observed in some of our RTS PDX models. This suggests that lncRNAs may regulate gene fusions that are a hallmark of many neoplasms.

Several potential limitations for our study should be noted. First, radiation therapy selection occurred in a heterotopic tumor location and within immunocompromised (athymic) mice. While prior data suggest that implanting tumors in subcutaneous sites or even culturing in serum-free neural stem cell media and later reimplanting in orthotopic sites does not compromise model fidelity (50, 51), there is concern that subcutaneous selection might influence tumor evolution or restrict tumor diversity. While our RNA-Seq data mapped highly to humans, suggesting a limited number of murine cells in the bulk sample, we did perform DE for mouse transcripts and found that mitochondrial genes were significantly decreased in RTS lines compared with RTU lines (Supplemental Figure 6), suggesting that the radiation selection of GBM tumors alters the recruitment of nontumor stromal cells and that these nontumor stromal cells are functionally unique from the cells recruited by the parental tumor. Another potential limitation is the lack of detailed patient clinical characteristics for the original tumors used to generate the PDXs. Our molecular data, however, suggest that our models retain diversity and display features that mirror recurrent clinical specimens. Future work will confirm the master regulatory potential of the numerous lncRNA targets that were identified, particularly in their connection with DDR and kinase activation. Moreover, most SMIs effectively target multiple kinases. As such, the relative contribution of individual kinases associated with the RTS phenotype is unclear based on drug treatment (or response) alone. Additional studies are needed to pinpoint the key kinase regulators in the RTS tumors, including in vivo experimentation.

To our knowledge, our in vivo RTS GBM PDX models are unique in the field. The differential enrichment in vivo of GBM kinase signaling is recapitulated in vitro, allowing for this model system’s versatility in the study of GBM tumor recurrence. Our in silico lncRNA analysis pipeline has identified known and potentially novel lncRNAs associated with therapy resistance. We have presented evidence that links several lncRNAs to DEGs in a variety of differentially regulated key GBM molecular pathways. Our results are consistent with the known regulatory roles of lncRNAs and may suggest this class of molecules as potential therapeutic targets in the treatment of recurrent, intractable GBM as well as in other cancers. We have also shown that our integrated workflow is able to reveal actionable lncRNA-related pathway targets using SMIs that have efficacy in recurrent, therapy-selected tumors. The combination of these RTS models with matched global kinomic and transcriptomic analysis represents a likely novel preclinical method for identifying druggable targets in intractable, recurrent GBM tumors.

## Methods

### PDX generation

PDXs were established from either primary or recurrent GBM surgical specimens obtained from the Brain Tumor Tissue Core of UAB under IRB and IACUC approval. These PDXs were established by injecting tumor chunks from patient tumors into the flank of athymic nude mice (Charles River Laboratories). These tumors were then serially passaged in mice with periodic cryopreservation of tumor chunks at various passages for future cultivation and study. Additional PDXs were acquired from CD James (Northwestern University, Chicago, Illinois, USA) and JN Sarkaria (Mayo Clinic, Rochester, Minnesota, USA), including 4 tumors that are isogenic to 4 of these parental PDXs from Sarkaria based on selection of TMZ-resistant tumors from previously TMZ-sensitive PDXs (52). A total of 20 GBM PDXs were initially included in our screenings for this study.

### PDX in vivo radiation screening and selection

Radiation screening and selection were performed in heterotopic PDXs cultivated in athymic nude mice. Ionizing radiation was delivered using an X-Rad 320 (Precision X-Ray Irradiation) and measured using a dosimeter placed in the treatment field. Heterotopically tumor-implanted mice (*n* = 5 per group) were anesthetized and administered 6 fractions of 2 Gy each over a period of 2 weeks with doses delivered on Monday, Wednesday, and Friday. Radiation administration was initiated when the average tumor volume of the 5 mice was measured to be approximately 200 cc. Tumor volume was measured using tissue calipers a minimum of 3 times per week during and following the treatment period. Tumor volume was calculated using the modified ellipsoid formula ½ (length × width^2^) (53, 54). The endpoint of the assay was defined by the time in days it took for the tumor to double in volume from its initial volume on the first day of irradiation. Tumor doubling time less than 20 days was defined as resistant to therapy and longer than 20 days was sensitive. Tumors found to be initially radiation sensitive then underwent serial selection for 6–8 rounds of radiation therapy. The fastest growing tumor from each previous round was harvested and implanted heterotopically into nude mice (*n* = 2) for the subsequent radiation round. Highly sensitive tumors that were “cured” by the standard 12 Gy treatment received only 3 × 2 Gy fractions for the initial 2 rounds of selection, after which the dosage was returned to the standard course. After the selection rounds were completed, repeat screening with 5 mice per group was performed to confirm the resistant, RTS phenotype.

### Orthotopic PDXs

Orthotopic PDXs were established by stereotactic intracranial injection of 300,000 tumor cells, dissociated from flank tumors, suspended in 5 μL of 5% methylcellulose in DMEM (32). Tumors from control mice were resected and snap-frozen in liquid nitrogen for later molecular analysis.

### WES

Genomic DNA was extracted from flash-frozen tumors using the QIAGEN DNeasy Blood & Tissue Kit. Sequencing libraries were prepared using the Illumina TruSeq DNA Exome kit, prior to pooling in groups of 12 and sequencing using a NextSeq 550 (Illumina) with a NextSeq 500/550 High Output Kit v2.5 (150 cycles) using paired-end settings (2 × 75).

#### SNP calls.

Raw paired-end exome reads were mapped to a human (hg38) and mouse (mm10) combined reference genome using TopHat (version 2.1), which uses bowtie2 (version 2.3.3) as its underlying short read mapper. Next, an mpileup file was created using bcftools (version 1.6). bcftools was then used to call variants. Vcftools.pl varFilter was then used to filter the calls by sequence read depth (minimum depth 500 nucleotides across all samples). The called SNPs that passed this quality thresholding were then used as input to SNPRelate (version 1.26.0) within R (version 4.1.2). The first 10 principal components were extracted from SNPRelate and were then used to perform MDS. A distance matrix was calculated between each sample, and a plot was made (using corrplot package version 0.92) with 1 – (Euclidean distance) of the MDS plot.

#### Copy number alteration calls.

Raw paired-end exome reads were mapped to a human (hg38) and mouse (mm10) combined reference genome using bowtie2 (version 2.3.3). Next, raw reads were aggregated using patternCNV script bam2wig.sh. PatternCNV pipeline was run per manual with default parameters. Log_2_ fold change calls data were extracted from patternCNV workflow. Data were concatenated and imported into R for data visualization. Exon-level data was summarized by gene using dplyr package. Pheatmap package was used to create a heatmap. In addition, genome-wide plots were visually examined for recurrent large copy number alterations in the individual RT pairs. Two RTS lines had large chromosome segment amplifications in copy number at chromosome 12q. A zoomed-in view of this locus is shown in the parental line and the RTS lines for the 2 affected PDXs (Figure 2B).

### RNA-Seq

Orthotopic tumors were resected from mouse brains, divided into 2 halves that were placed in sterile screw-cap vials (Sarstedt), and snap-frozen in liquid nitrogen. Samples were stored at –70°C until used. One half of the tumor was used for RNA isolation and the other for protein lysate. RNA extraction was performed using the Norgen Animal Tissue RNA extraction kit following the kit instructions. Snap-frozen tissue was first ground into a powder using a mortar and pestle, then processed according to the Norgen kit. RNA was quantified using a NanoDrop (Thermo Fisher Scientific), and 50 μg of RNA was sub-aliquoted for RNA-Seq. RNA-Seq was performed by GENEWIZ. RNA integrity and fragment size were tested using RNA ScreenTape (Agilent Technologies TapeStation) with average RNA integrity numbers of 8.5 and average fragment length around 4500 nt. The samples were then depleted of rRNA before conducting stranded library preparation. Sequencing using biological triplicates for each sample was performed on an Illumina HiSeq 2000 using 150 bp paired-end reads with a median of around 72 million reads per sample. FastQC reports were generated pretrimming and posttrimming of low-quality bases (Phred score < 20) and Illumina adapter sequences using trim-galore-0.4.5. Quasi-mapping and quantification were performed using Salmon-0.12.0 (with --gencode and -k 31 flags for index generation and -l ISR, --gcBias, and --validateMappings flags for quantification) against the Gencode GRCh38.p12 release 31 reference transcriptome. A multiQC report was then generated including the use of FastQ Screen (v0.11.2 with dependencies to bowtie2 v2.3.3 and SAMtools v1.6) to identify the proportions of mouse to human transcripts detected. Three replicates were excluded from further analysis due to high mouse transcript contamination. We retrospectively determined from resection notes that these high mouse content tumors were highly infiltrative and hemorrhagic upon resection. A tx2gene table was then generated from a TxDB object using Bioconductor genomicfeatures-1.32.3. This table and the quant.sf files from Salmon were then read using tximport (v1.16.1), biomaRt (v2.44.4), and DESeq2 (v1.28.1) to generate normalized expression tables used in further in silico analysis.

### Kinomic analysis

Kinome profiling was performed in the UAB Kinome Core as done previously (55, 56). Another portion of the same snap-frozen orthotopic tumor was lysed with pestle grinding for 30 minutes in M-PER lysis buffer (Pierce) at 4°C to extract protein (Bradford assay quantified) for kinomic analysis. Samples (15 μg PTK, 2 μg STK per array), in biological triplicate, were processed and run on the tyrosine (PTK; 86402 PamChips, PamGene) or serine/threonine (STK; 97102 PamChips) arrays, testing phosphorylation kinetically against 196 (PTK) or 144 (STK) 12 to 15 amino acid substrate targets on the PamStation12 (PamGene). Phosphorylation of peptides was measured over 154 (PTK) or 164 (STK) cycles, with exposures from 10 to 200 ms that were integrated into slopes, multiplied by 100, and log_2_-transformed in BioNavigator (v6.3) (PamGene). Mean peptide phosphorylation per PDX (i.e., mean of replicates) was used for heatmap clustering (unsupervised geometric means-distance), and change per PDX pair per barcode was used for upstream kinase prediction (UpKin PamApp; PTK v6.0, STK v6.0) and for PCA (Shiny PamApp). Kinases with a mean final score > 2.0 or mean kinases statistic > 5.0 were retained.

### In vitro drug response viability assay

PDXs were propagated as neurospheres, cultured in PDX media (DMEM/F12) (50/50 with 2% B27, 20 ng/mL EGF, 20 ng/mL basic FGF, 1% sodium pyruvate, 1% penicillin and streptomycin) similar to our prior reports (57). Neurospheres were dissociated for 20 minutes with Accutase (Innovative Cell Technologies) at 37°C, viability-quantified with trypan blue utilizing the Countess II (Thermo Fisher Scientific), and plated at 500–1500 viable cells per 100 μL for 24 hours prior to treatment with indicated doses of brigatinib and sitravatinib (Selleckchem). After 7 days, CTG reagent (Promega) was added at 20 μL per well for 30 minutes at 37°C, prior to reading luminescence on the BioTek Synergy H1 (Agilent). Raw values were corrected to untreated (DMSO) control.

### Antibody-based phospho-kinase array analysis

PDX cells were maintained in neurobasal media as described previously (57). Cells were lifted from Geltrex (Gibco) with Accutase (Innovative Cell Technologies), lysates were collected with supplied reagents, and phospho-specific protein abundance was quantified using Phospho-Kinase Arrays (ARY003B, R&D Systems). Cells were collected according to the manufacturer’s protocol, with protease and phosphatase inhibitors and protein quantified as in the kinomics methods section. A total of 300 micrograms of protein were loaded per array, and probed overnight, prior to secondary application for 4 hours and chemiluminescent (PerkinElmer Western Lightning Plus) development and film scanning. Image analysis was conducted in ImageJ (v1.53p, NIH) with background removal (rolling ball, 150 pixels, 1200 dpi image) prior to image inversion and manual spot selection and intensity quantitation on identical sized regions of interest. All spots were quantified in duplicate. Spots with a mean intensity change of greater than 25% between groups were heatmapped by percentage change using GraphPad Prism 9.

### In silico analysis

The DESeq2 R package was used for normalization, clustering, and DE. PCAs were performed using the R stats and factoextra packages. DGCA was performed using the DGCA R package (v1.0.2) with prefiltering of zero counts and a filter based on empirical Bayes statistics for DEGs between RTU and RTS replicates for each sample. The combined normalized expression table and expression table based on pairwise DEG results for each sample were used as an input to FastEMC (v0.0.6), which selected and sorted the genes that best discriminated between the sensitive and the resistant phenotype. Normalized expression was in parallel used as the input for the WGCNA R package (v1.69), which selected gene modules that distinguished between and within samples. The modules within samples were analyzed with BEERE to generate interaction networks, which were then entered in Gene Terrain to visualize the differential utilization of genes within the modules between RTU and RTS PDXs. A table of cDNA was converted into FASTA format for lncRNAs, and coding genes were identified by DEG, DGCA, and FastEMC using the biomaRt package. FASTAs for lncRNAs and coding genes were inputs for ASSA (v1.00) to identify RNA:RNA predicted interactions. FASTAs for the lncRNA and the Ensembl Regulatory Build (58) were inputs for Triplexator in order to identify lncRNA:DNA triple helix sites. Triplexator flags –m R,Y,M,P,A; –v -of 0; and –rm 2 were used. Results were filtered to remove any results that contained errors. LncRNA:RNA and lncRNA:DNA binding results were visualized using Circos. Bedtools (v2.29.2) was then used to search 20 kb upstream and downstream from triple helix sites to identify genes proximal to these potential binding sites. Genes that were both proximal to the lncRNA binding sites and identified as DEGs were considered purported lncRNA cis-regulatory targets. The R corrr (v0.4.2) package was used to find correlations between the expression of lncRNAs and the proximal genes. Correlations were also calculated for pairwise and global differentially regulated genes that intersected curated gene sets for DDR, stemness, chromatin remodeling, and cell cycle progression, among others. Genes of interest from DEGs, DGCA, FastEMC, ASSA, and Triplexator/BedTools were then input into PAGER and GSEA (59, 60) for gene set enrichment analysis. Key terms from PAGER were then combined with the lists of differentially regulated genes as input for BEERE, which constructed gene:semantic interaction networks. The intersection of DEGs from each PDX pair with lncRNAs and their proximal genes was used as input to WIPER, which identified key gene pair relationships and generated lncRNA:mRNA transcript interaction networks. In parallel, upstream kinomics data were combined with lncRNA proximal genes of interest using MetaCore/GeneGo (Clarivate Analytics) for integrated pathway analysis.

### Inherent radiation resistance analysis

To assess the transcriptomic differences between inherently resistant and sensitive tumors, we performed differential expression analysis between these 2 groups (*n* = 7 sensitive, *n* = 6 resistant) using Affymetrix array data (Whole Transcript Human Exon 1.0 ST). Data were quantile-normalized together with the downloaded Affymetrix Human Exon 1.0 ST data of the core TCGA samples (61). Data were then summarized into expression levels for each gene using the pipeline previously described (62). PDX samples were run in triplicate and collapsed using the average expression. The EdgeR and limma R packages were used to perform the differential expression analysis. Samples were grouped into a DGEList object, and then normalization factors were calculated. The model used for analysis was ~0+PDX_ID+RT_Status to account for differences in radiation status as well as tumor-specific baseline differences. The dispersion was then estimated using the estimateDisp() function. We then tested the differences between inherently resistant and sensitive tumors using the exactTest() function. A Benjamini-Hochberg–adjusted *P* value of 0.05 was used as the significance threshold. Overrepresentation analysis was then performed in WebGestalt using available Gene Ontology and pathway databases. Enrichment analysis was also performed using the Broad GSEA program using the default settings and a combination of MSigDB sets as well as our custom gene sets.

### Figure preparation

Figures 1–4 and 6, along with the graphical abstract, were created with BioRender.com under its academic license terms.

### Data and resource availability

Requests for data, resources/reagents, software, and further information should be directed to the corresponding author. WES data can be downloaded using accession number PRJNA847439 (https://www.ncbi.nlm.nih.gov/bioproject/PRJNA847439/) from National Center for Biotechnology Information’s (NCBI) BioProject. The RNA-Seq data have been deposited in NCBI’s Gene Expression Omnibus (GEO) and are accessible through GEO Series accession number GSE206225 (https://www.ncbi.nlm.nih.gov/geo/query/acc.cgi?acc=GSE206225). Detailed in silico methods are included in the Supplemental Methods.

### Statistics

*P* values for differential expression were calculated using Fisher’s exact test and then adjusted using the Benjamini-Hochberg procedure. FDRs were used for all GSEA, ORA, and PAGER results. In vitro growth assays including comparisons of tumor median doubling times were evaluated for significance using a standard 2-tailed *t* test. Comparisons of Kaplan-Meier endpoint probability traces were made using log-rank tests. *P* values less than 0.05 were considered significant.

### Study approval

PDXs were established from either primary or recurrent GBM surgical specimens obtained from the Brain Tumor Tissue Core of UAB under IRB approval X050415007 and IACUC approval 21435.

## Author contributions

CTS, GYG, and CDW conceived the study; CTS, GYG, and CDW developed experimental design; CTS wrote the first draft; JCA, ZY, TN, NJE, CPL, JW, JRR, CX, FMM, ESY, ABH, CRM, XC, JYC, GYG, and CDW edited the manuscript; CTS, JCA, NJE, CX, FMM, ESY, REB, and HA collected data; CTS, JCA, ZY, TN, JW, JRR, KJL, XC, and CDW analyzed data; ABH, CRM, JYC, GYG, and CDW acquired funding.

## Supplementary Material

Supplemental data

Supplemental data set 1

Supplemental data set 2

Supplemental data set 3

Supplemental data set 4

## Version 1 07/19/2022

In-Press Preview

## Version 2 08/22/2022

Electronic publication

## References

[1] Von Rosenstiel C (2020). Correlation of the quantitative level of MGMT promoter methylation and overall survival in primary diagnosed glioblastomas using the quantitative MethyQESD method. J Clin Pathol.

[2] Stupp R (2005). Radiotherapy plus concomitant and adjuvant temozolomide for glioblastoma. N Engl J Med.

[3] Hegi ME (2005). MGMT gene silencing and benefit from temozolomide in glioblastoma. N Engl J Med.

[4] Szopa W (2017). Diagnostic and therapeutic biomarkers in glioblastoma: current status and future perspectives. Biomed Res Int.

[5] Tang Z (2022). Radioresistance and transcriptional reprograming of invasive glioblastoma cells. Int J Radiat Oncol Biol Phys.

[6] Wu Q (2021). The impact of epigenetic modifications on adaptive resistance evolution in glioblastoma. Int J Mol Sci.

[7] Nicholson JG, Fine HA (2021). Diffuse glioma heterogeneity and its therapeutic implications. Cancer Discov.

[8] Berg TJ (2021). The irradiated brain microenvironment supports glioma stemness and survival via astrocyte-derived transglutaminase 2. Cancer Res.

[9] Fletcher-Sananikone E (2021). Elimination of radiation-induced senescence in the brain tumor microenvironment attenuates glioblastoma recurrence. Cancer Res.

[10] Kang H (2021). Downregulated CLIP3 induces radioresistance by enhancing stemness and glycolytic flux in glioblastoma. J Exp Clin Cancer Res.

[11] Osuka S (2021). N-cadherin upregulation mediates adaptive radioresistance in glioblastoma. J Clin Invest.

[12] Stackhouse CT (2021). Cancer explant models. Curr Top Microbiol Immunol.

[13] Biau J (2017). Predictive biomarkers of resistance to hypofractionated radiotherapy in high grade glioma. Radiat Oncol.

[14] Barthel FP (2019). Longitudinal molecular trajectories of diffuse glioma in adults. Nature.

[15] Korber V (2019). Evolutionary trajectories of IDH ^WT^ glioblastomas reveal a common path of early tumorigenesis instigated years ahead of initial diagnosis. Cancer Cell.

[16] Bhat SA (2016). Long non-coding RNAs: mechanism of action and functional utility. Noncoding RNA Res.

[17] Stackhouse CT (2020). Exploring the roles of lncRNAs in GBM pathophysiology and their therapeutic potential. Cells.

[18] Carlevaro-Fita J (2020). Cancer LncRNA census reveals evidence for deep functional conservation of long noncoding RNAs in tumorigenesis. Commun Biol.

[19] Patro R (2017). Salmon provides fast and bias-aware quantification of transcript expression. Nat Methods.

[20] Antonov I (2018). ASSA: fast identification of statistically significant interactions between long RNAs. J Bioinform Comput Biol.

[21] Buske FA (2012). Triplexator: detecting nucleic acid triple helices in genomic and transcriptomic data. Genome Res.

[22] McKenzie AT (2016). DGCA: a comprehensive R package for differential gene correlation analysis. BMC Syst Biol.

[23] Langfelder P, Horvath S (2008). WGCNA: an R package for weighted correlation network analysis. BMC Bioinformatics.

[24] Stackhouse CT (2019). A novel assay for profiling GBM cancer model heterogeneity and drug screening. Cells.

[25] Yue Z (2015). PAGER: constructing PAGs and new PAG-PAG relationships for network biology. Bioinformatics.

[26] Yue Z (2019). BEERE: a web server for biomedical entity expansion, ranking and explorations. Nucleic Acids Res.

[27] Bhullar KS (2018). Kinase-targeted cancer therapies: progress, challenges and future directions. Mol Cancer.

[28] Kim RK (2015). Radiation promotes malignant phenotypes through SRC in breast cancer cells. Cancer Sci.

[29] Jarboe JS (2012). Kinomic profiling approach identifies Trk as a novel radiation modulator. Radiother Oncol.

[30] Dolan M (2019). Enhanced efficacy of sitravatinib in metastatic models of antiangiogenic therapy resistance. PLoS One.

[31] Camidge DR (2018). Exploratory analysis of brigatinib activity in patients with anaplastic lymphoma kinase-positive non-small-cell lung cancer and brain metastases in two clinical trials. J Clin Oncol.

[32] Giannini C (2005). Patient tumor EGFR and PDGFRA gene amplifications retained in an invasive intracranial xenograft model of glioblastoma multiforme. Neuro Oncol.

[33] Fischer U (2010). Amplicons on chromosome 12q13-21 in glioblastoma recurrences. Int J Cancer.

[34] Huber RM (2020). Brigatinib in crizotinib-refractory ALK+ NSCLC: 2-year follow-up on systemic and intracranial outcomes in the phase 2 ALTA trial. J Thorac Oncol.

[35] Du W (2018). Sitravatinib potentiates immune checkpoint blockade in refractory cancer models. JCI Insight.

[36] Kenchappa RS (2021). Protein kinase C_ι_ and SRC signaling define reciprocally related subgroups of glioblastoma with distinct therapeutic vulnerabilities. Cell Rep.

[37] D’Andrea AD (2010). Susceptibility pathways in Fanconi’s anemia and breast cancer. N Engl J Med.

[38] Castillo P (2011). Coordinated action of the Fanconi anemia and ataxia telangiectasia pathways in response to oxidative damage. DNA Repair (Amst).

[39] Sobeck A (2009). The Fanconi anemia protein FANCM is controlled by FANCD2 and the ATR/ATM pathways. J Biol Chem.

[40] Chun J (2013). Rad51 paralog complexes BCDX2 and CX3 act at different stages in the BRCA1-BRCA2-dependent homologous recombination pathway. Mol Cell Biol.

[41] Onagoruwa OT (2020). Oncogenic role of PVT1 and therapeutic implications. Front Oncol.

[42] Wang XD (2019). Functional role of long non-coding RNA CASC19/miR-140-5p/CEMIP axis in colorectal cancer progression in vitro. World J Gastroenterol.

[43] Hu Y (2017). Candidate tumor suppressor ZNF154 suppresses invasion and metastasis in NPC by inhibiting the EMT via Wnt/β-catenin signalling. Oncotarget.

[44] Zhang W (2018). ZNF154 is a promising diagnosis biomarker and predicts biochemical recurrence in prostate cancer. Gene.

[45] Jeong EG (2008). Somatic mutations of JAK1 and JAK3 in acute leukemias and solid cancers. Clin Cancer Res.

[46] Nairismägi ML (2018). Oncogenic activation of JAK3-STAT signaling confers clinical sensitivity to PRN371, a novel selective and potent JAK3 inhibitor, in natural killer/T-cell lymphoma. Leukemia.

[47] Yang Z (2019). SOX11: friend or foe in tumor prevention and carcinogenesis?. Ther Adv Med Oncol.

[48] Sturm D (2014). Paediatric and adult glioblastoma: multiform (epi)genomic culprits emerge. Nat Rev Cancer.

[49] Gambella A (2020). NTRK fusions in central nervous system tumors: a rare, but worthy target. Int J Mol Sci.

[50] Haddad AF (2021). Mouse models of glioblastoma for the evaluation of novel therapeutic strategies. Neurooncol Adv.

[51] Jacob F (2020). A patient-derived glioblastoma organoid model and biobank recapitulates inter- and intra-tumoral heterogeneity. Cell.

[52] Kitange GJ (2012). Inhibition of histone deacetylation potentiates the evolution of acquired temozolomide resistance linked to MGMT upregulation in glioblastoma xenografts. Clin Cancer Res.

[53] Euhus DM (1986). Tumor measurement in the nude mouse. J Surg Oncol.

[54] Tomayko MM, Reynolds CP (1989). Determination of subcutaneous tumor size in athymic (nude) mice. Cancer Chemother Pharmacol.

[55] Chandrashekar DS (2020). Therapeutically actionable PAK4 is amplified, overexpressed, and involved in bladder cancer progression. Oncogene.

[56] Ibrahim AN (2019). Intratumoral spatial heterogeneity of BTK kinomic activity dictates distinct therapeutic response within a single glioblastoma tumor. J Neurosurg.

[57] Eustace NJ (2020). A cell-penetrating MARCKS mimetic selectively triggers cytolytic death in glioblastoma. Oncogene.

[58] Zerbino DR (2015). The ensembl regulatory build. Genome Biol.

[59] Mootha VK (2003). PGC-1alpha-responsive genes involved in oxidative phosphorylation are coordinately downregulated in human diabetes. Nat Genet.

[60] Subramanian A (2005). Gene set enrichment analysis: a knowledge-based approach for interpreting genome-wide expression profiles. Proc Natl Acad Sci U S A.

[61] Verhaak RG (2010). Integrated genomic analysis identifies clinically relevant subtypes of glioblastoma characterized by abnormalities in PDGFRA, IDH1, EGFR, and NF1. Cancer Cell.

[62] Lockstone HE (2011). Exon array data analysis using Affymetrix power tools and R statistical software. Brief Bioinform.

