# Supplementary material for: An in vivo model of glioblastoma radiation resistance identifies long noncoding RNAs and targetable kinases
Source: JCI Insight. 2022 Aug 22;7(16):e148717. doi: 10.1172/jci.insight.148717 (PMC9462495; doi:10.1172/jci.insight.148717)
Supplement: Supplemental data [file jciinsight-7-148717-s017.pdf]

Supplementary Information:

Overview

The pipeline begins with deep sequencing of total RNA from orthotopic xenograft tumors with a confirmed phenotype. The raw sequencing files are then processed using the Salmon pseudo-aligner which multiple benchmarks have revealed has better performance at calling lncRNAs than other methods such as STAR (1, 2). Next, a machine learning algorithm we developed (3) to call features significantly related to a given phenotype is employed in parallel with differential expression analysis using DESeq2 (4) and differential correlation analysis using DGCA (5). The sequences of all isoforms of the differentially regulated lncRNAs associated with the observed phenotype are then tested with two thermodynamics-based programs, Triplexator (6) and ASSA (7) to evaluate the nucleic acid binding potential of these transcripts. Triplexator identifies both the regions on the transcript of interest which possess DNA hybridization potential and the genomic loci in the human regulatory genome reference that these sites can form triple helices with. We then obtain lists of genes proximal to these lncRNA:DNA hybrid sites and correlate the expression of proximal genes with the lncRNAs. We then construct semantic networks and perform gene set enrichment analysis (GSEA) (8) to reveal the relationships between lncRNA expression and cancer-relevant networks.

In silico analysis

Feature Selection:

The DESeq2 R package was used for normalization, clustering, and DEG. A Benjamini-Hochberg (BH)-adjusted p-value of 0.05 was used as the significance threshold. PCAs were performed using the R base stats and factoextra (v1.0.7) packages. DGCA was performed using the DGCA R package (v1.0.2) with pre-filtering of zero counts and a filter based on empirical Bayes statistics for DEG between RTU and RTS replicates for each sample. The normalized expression table was filtered using the combined list of DEG and DGCA results for each sample (filtered normalized counts table). This table was used as an input to FastEMC (v0.0.6) which selected and sorted the genes which best discriminated between the sensitive and resistant phenotype (3). The top 100 predictive features (genes) were retained for downstream analysis. The FastEMC algorithm has since been updated utilizing the scikit-learn ML package in python (https://rowland-208.github.io/ivygap/).

Gene-module selection and visualization:

The unfiltered normalized expression table was in parallel used as the input for the WGCNA R package (v1.69) which selected gene modules that distinguished between and within samples. The modules within samples were analyzed with BEERE to generate interaction networks which were then input in Gene Terrain to visualize the differential utilization of genes within the modules between RTU and RTS PDX(9, 10).

Nucleic acid binding prediction:

A table of cDNA was converted into FASTA format for lncRNAs and coding genes identified by DEG, DGCA, and FastEMC using the biomaRt package. FASTAs for lncRNAs and coding genes were input into ASSA (v1.00) to identify RNA:RNA predicted interactions. ASSA results were filtered to remove duplicates and using an adjusted p-value cutoff of 0.0001 or less. FASTAs for the lncRNA and the Ensembl Regulatory Build (11) were also input into Triplexator in order to identify lncRNA:DNA triple helix sites. Triplexator flags –m R,Y,M,P,A; –v -of 0; and –rm 2 were used. Results were filtered manually in Excel to remove any results which contained errors and duplicates were removed. Chromosomal coordinates were then extracted into a new BED file (https://m.ensembl.org/info/website/upload/bed.html). This file was used to visualize lncRNA:RNA and lncRNA:DNA binding results using Circos.

Identification of purported lncRNA cis-regulatory elements:

The BED file was used as input to BedTools (v2.29.2) to search 20kb upstream and downstream (40kb window) from each triple helix site to identify genes proximal to these potential binding sites. The lists of lncRNA binding site proximal genes were compared against the global and pairwise DEG lists in R to find the union of significantly DEGs proximal to lncRNA binding sites. Genes that were both proximal to the lncRNA binding sites and identified as DEGs were considered as purported lncRNA cis-regulatory targets. The R corrr (v0.4.2) package was used to find correlations between the expression of lncRNAs and the proximal genes. Correlations were also calculated for pairwise and global differentially regulated genes that intersected curated gene sets for DDR, stemness, chromatin remodeling, cell cycle progression, among others. Genes of interest from DEG, DGCA, FastEMC, ASSA, and Triplexator/BedTools were then input into PAGER (12) for enrichment analysis. Key terms from PAGER were then combined with the lists of differentially regulated genes as input for BEERE which constructed gene:semantic interaction networks. The intersection of DEGs from each PDX pair with lncRNAs and their proximal genes were used as input to WIPER (13) which identified key gene pair relationships and generated lncRNA:mRNA transcript interaction networks.

Potential lncRNA regulation of DDR gene sets:

The unfiltered normalized expression table was input to GSEA (8) with custom gene sets (.gmt files) including the curated gene signatures of interest. The fmsb (v0.7.3) R package radarchart() function was used to generate radar plots for each pairwise comparison and global RTU vs RTS plotting the normalized enrichment scores (NES) from GSEA. Significantly DEGs that were proximal to lncRNA-binding sites of differentially regulated lncRNAs in each comparison were compared to the DDR gene-set lists. The genes which overlapped were added to the radar plots with the log-fold change and the sign of the correlation (corrr package) of that gene's expression with the expression of the lncRNA which binds proximal to that gene's locus.

Integration of lncRNA-regulated DEGs with differential kinase activity:

Upstream kinomics data was combined with lncRNA proximal genes of interest using MetaCore/GeneGo (Clarivate Analytics, Philadelphia, PA) for integrated pathway analysis. These integrated pathways are constructed for each PDX pair including the significant kinases

differentially regulated between RTU and RTS along with related pathway enrichment based on lncRNA binding proximal genes selected by above mentioned methods.

Additional Differential Expression Approaches: Several approaches were employed to complement the standard DE analysis by identifying combinations of features which are highly predictive, in the case of machine learning (ML) or which are differentially regulated between phenotypes in the case of differential gene correlation analysis (DGCA). Transcriptomic features which discriminated RTU from RTS samples were selected globally and pairwise using Fast Exponential Monte Carlo or FastEMC (3). This unbiased, ML approach ranks combinations of transcriptomic features in order of their predictive power in distinguishing between RTU and RTS PDX. The top 250 predictive features from FastEMC, including 29 lncRNAs and 10 pseudogenes, exhibited overlap with global DEGs, pairwise DEGs, and gene signatures for DDR, stemness, chromatin remodeling, and cell cycle progression. Several of the lncRNAs identified by FastEMC have predicted DNA binding sites proximal to significantly DEGs (**Figure 3** and **Supplementary Figure S3**). DGCA (5) was employed to identify the top differentially correlated gene pairs between RTU and RTS PDX. The top 1000 differentially correlated gene pairs along with significantly DEGs and the top 250 predictive genes from FastEMC were combined for further downstream analysis.

Correlations of lncRNA and significantly altered DEGs: After confirming the non-coding potential of the lncRNA targets using two inspection strategies (Coding Potential Calculator 2.0 found at http://cpc2.gao-lab.org/ and AnnoLnc 2.0 found at http://annolnc.gao-lab.org/), we performed correlation studies with the significantly altered DEG's. The X1516 pair, DUXAP9 and DUXAP10 were positively correlated with chromatin remodeling gene RBBP7 while negatively correlated with HDAC9 and RERE (**Supplementary Figure S3**). In the X1153 pair, ZFAS1, SAMMSON, and SOX2-OT showed a mixture of positive and negative correlations with stemness-related COVL, PTPRZ1, and MAPRE2 genes (**Supplementary Figure S3**). In the JX12T pair, ZFAS1 also showed a positive regulation with MAPRE2 while simultaneously having a negative correlation with cell-cycle gene RFC3 (**Supplementary Figure S3**). Across multiple lncRNAs, the forkhead box P1 (FOXP1) transcription factor is targeted both directly and indirectly. Correlation with proximal targets of FOXP1 reveals overlap with predictive features from FastEMC as well as with stemness, chromatin remodeling, and homologous recombination signatures. Additionally, the lncRNA AF106564.1 showed a strong positive correlation with the expression of AC058822.1 (ENSG00000282278), the novel FIP1L1-PDGFRA fusion transcript as well as with NTRK3 (ENSG00000140538) which is a putative growth factor for CNS tumors and is also a component of multiple oncogenic fusion products (14).

Network Analysis Reveals Differential Pathway Enrichment in RTS PDX: Pathway, gene set, gene module, and gene network level analyses were completed using GSEA (8), WebGestalt (15), Pathway, Annotated-list, and Gene-signature Electronic Repository (PAGER) (12), Weighted Gene Correlation Network Analysis (WGCNA) (16), Gene Terrain (10), Biomedical Entity Expansion, Ranking, and Exploration (BEERE) (9), and Weighted In-Path Edge Ranking for biomolecular association networks (WIPER) (13). The WGCNA ML approach identified 7 clusters of gene modules which distinctly identify the PDX samples by patient tumor of origin (**Figure S4A**). Gene interaction networks were formed for each of these gene module clusters

using BEERE. Differential regulation of these networks is observed between RTU and RTS PDX within each pair using Gene Terrain to overlay average gene expression on the gene network coordinates (**Figure S4B**). There are 160 significantly DEGs within the JX12T WGCNA gene modules with the majority of genes, including cell cycle related genes, upregulated in the RTU line (**Figure S4A, B**). Over-representation analysis (ORA) identifies chromosome localization and cell cycle processes as being enriched in the JX12T-related gene modules (**Figure S4C**). Actively dividing cells, such as those enriched in JX12T RTU tumors, would be expected to be more susceptible to radiation-induced DNA damage. The top GSEA result for the JX12T-related gene modules were a signature of genes under-expressed in stem cell-like cholangiocarcinoma, enriched (over-expressed) in the JX12T RTU line (**Figure S4D**). This represents an enrichment of genes in stem-like cells, which are thought to confer therapy resistance, in the JX12T RTS PDX.

<u>WIPER Networks</u>: In order to identify the potential regulatory impact of lncRNAs on gene networks at the epigenetic level, we compared RTU/RTS DEGs to the genes proximal (within 40kb window) to predicted lncRNA DNA binding sites **Supplementary Figure S2B**. WIPER analysis (**Supplementary File S2**) of combined global DEGs intersecting with lncRNA DNA binding site proximal gene targets revealed a SMAD3 centric network, indicating potential enrichment of TGFR-beta signaling. Gene networks were constructed for each PDX pair using pairwise differentially regulated lncRNAs and significantly differentially expressed genes. The lncRNA-DNA binding potential and proximal gene list were constructed for each PDX pair. This list was then compared to the list of pairwise significantly differentially expressed genes to find the union of the two sets. In the JX12T, JX14P, X1465, and X1516 pairs, WIPER identified several collagen-related genes as central to these networks. This aligns with PAGER, GSEA, and ORA results highlighting changes in focal adhesion and extracellular matrix remodeling. In JX14T, networks were centered on lncRNAs ZFAS1, DUXAP10, AUXG01000058.1, and SOX2-OT as well as centers around EGFR, PIK3R1, and SRC. JX39P networks were centered on SAMMSON, DUXAP10, ZFAS1, AUXG01000058.1, ERBB2, STAT1, and proto-oncogene MYC. X1465 and X1516 pairs had proto-oncogene FOS centric networks. EGFR, EGF, and ACTB were also central nodes in the X1465 network.

<u>Mouse transcriptome analysis</u>: Raw paired-end RNA-seq reads were mapped to a human (hg38) and mouse (mm10) combined reference genome using tophat2 which uses bowtie2 (version 2.3.3) as its short read mapper. HTSeq was used to quantify read counts for each gene locus for both the human and mouse genes. Separately, the human and mouse gene sets were imported into R. Library size normalization and differential gene expression analysis were performed DESeq package per user manual. Cell line was fit in the model to reduce variation due to individual level differences in gene expression and each gene was tested for changes as a function of RT selection, RTS versus RTU. Log-fold change for each gene was extracted from R in a rank list format and served as input for Gene Set Enrichment Analysis (GSEA) software (version 4.1.0). The pre-ranked gene set workflow of GSEA was performed for hallmark curated gene sets and positional gene sets. A false-discovery rate of 10% was used as a threshold for identifying altered gene sets. See **Supplementary Figure S6**.

Software List

Software (Versions)

- ASSA (v1.00)
- BedTools (v2.29.2)
- BEERE (http://discovery.informatics.uab.edu/BEERE/)
- BioNavigator (v6.3)
- Bowtie2 (v2.3.3)
- FastQC (v1.8.0)
- FastQ Screen (v0.11.2)
- GeneTerrain
- multiQC (v1.4)
- MetaCore/GeneGo
- PAGER (v2.0, http://discovery.informatics.uab.edu/PAGER/)
- Python (v2.7)
- WebGestAlt (http://www.webgestalt.org/)

Python Packages

  - FastEMC (v0.0.6)
  - Numpy (v1.19.4)
  - Pandas (v1.1.4)
  - Scikit-learn (v0.23.2)
  - Tqdm (v4.54.0)

- R (v4.0.2)

R Packages

  - Affy (v1.66.0)
  - BiocManager (v1.30.10)
  - biomaRt (v2.44.4)
  - Caret (v6.0-86)
  - clanC
  - Corrr (v0.4.2)
  - DESeq2 (v1.28.1)
  - DGCA (v1.0.2)

    - Dplyr (v1.0.2)
    - edgeR (v3.30.3)
    - Factoextra (v1.0.7)
    - FactoMineR (v2.3)
    - Fmsb (v0.7.3)
    - Genefilter (v1.70.0)
    - Genomicfeatures (v1.32.3)
    - Ggplot2 (v3.3.2)
    - Limma (v3.52.1)
    - pdfCluster (v1.0-3)
    - pheatmap (v1.0.12)
    - PoiClaClu (v1.0.2.1)
    - RColorBrewer (v1.1-2)
    - readR (v1.3.1)
    - Tximport (v1.16.1)
    - WGCNA (v1.69)
- Reference Genome: Ensembl GRCh37 version 97
- SAMtools (v1.6)
- Triplexator (v1.3.2)
- WIPER (http://discovery.informatics.uab.edu/WIPER/)

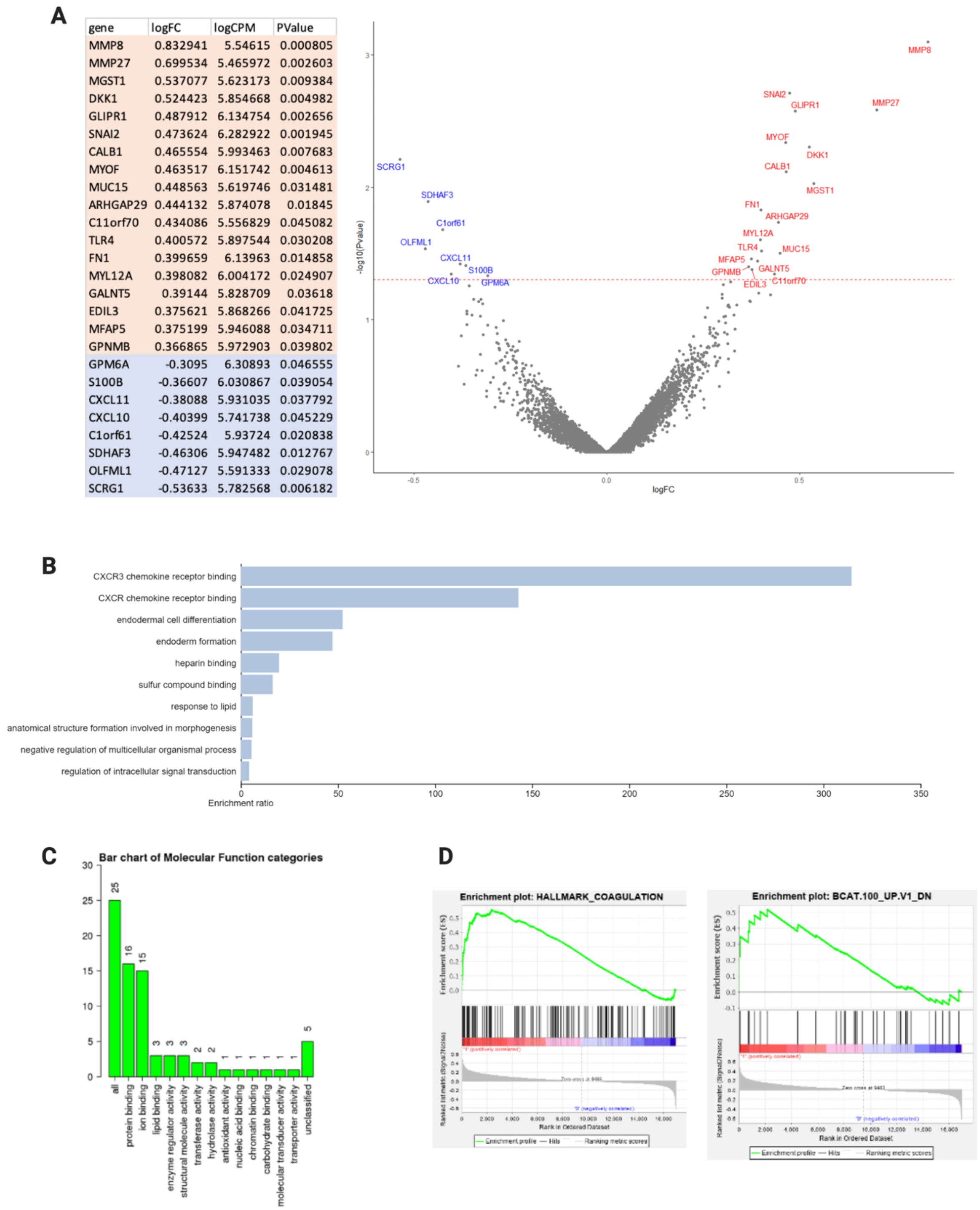

gene	logFC	logCPM	PValue
MMP8	0.832941	5.54615	0.000805
MMP27	0.699534	5.465972	0.002603
MGST1	0.537077	5.623173	0.009384
DKK1	0.524423	5.854668	0.004982
GLIPR1	0.487912	6.134754	0.002656
SNAI2	0.473624	6.282922	0.001945
CALB1	0.465554	5.993463	0.007683
MYOF	0.463517	6.151742	0.004613
MUC15	0.448563	5.619746	0.031481
ARHGAP29	0.444132	5.874078	0.01845
C11orf70	0.434086	5.556829	0.045082
TLR4	0.400572	5.897544	0.030208
FN1	0.399659	6.13963	0.014858
MYL12A	0.398082	6.004172	0.024907
GALNT5	0.39144	5.828709	0.03618
EDIL3	0.375621	5.868266	0.041725
MFAP5	0.375199	5.946088	0.034711
GPNMB	0.366865	5.972903	0.039802
GPM6A	-0.3095	6.30893	0.046555
S100B	-0.36607	6.030867	0.039054
CXCL11	-0.38088	5.931035	0.037792
CXCL10	-0.40399	5.741738	0.045229
C1orf61	-0.42524	5.93724	0.020838
SDHAF3	-0.46306	5.947482	0.012767
OLFML1	-0.47127	5.591333	0.029078
SCRG1	-0.53633	5.782568	0.006182

Supplementary Figure S1: Inherent radiation resistance differentially expressed gene analysis. A, Significantly altered genes within the inherently radiation resistant group are shown as log fold change with volcano plot. B, Over representation analysis of these 26 genes revealed no significant enrichment at an FDR of <.05, Top 10 results are shown. C, Enriched molecular function GO terms are shown for inherently resistant tumors. D, The top 2 enriched gene sets from GSEA using the MSigDB H and C6 sets are shown for inherently resistant PDX. Created with BioRender.

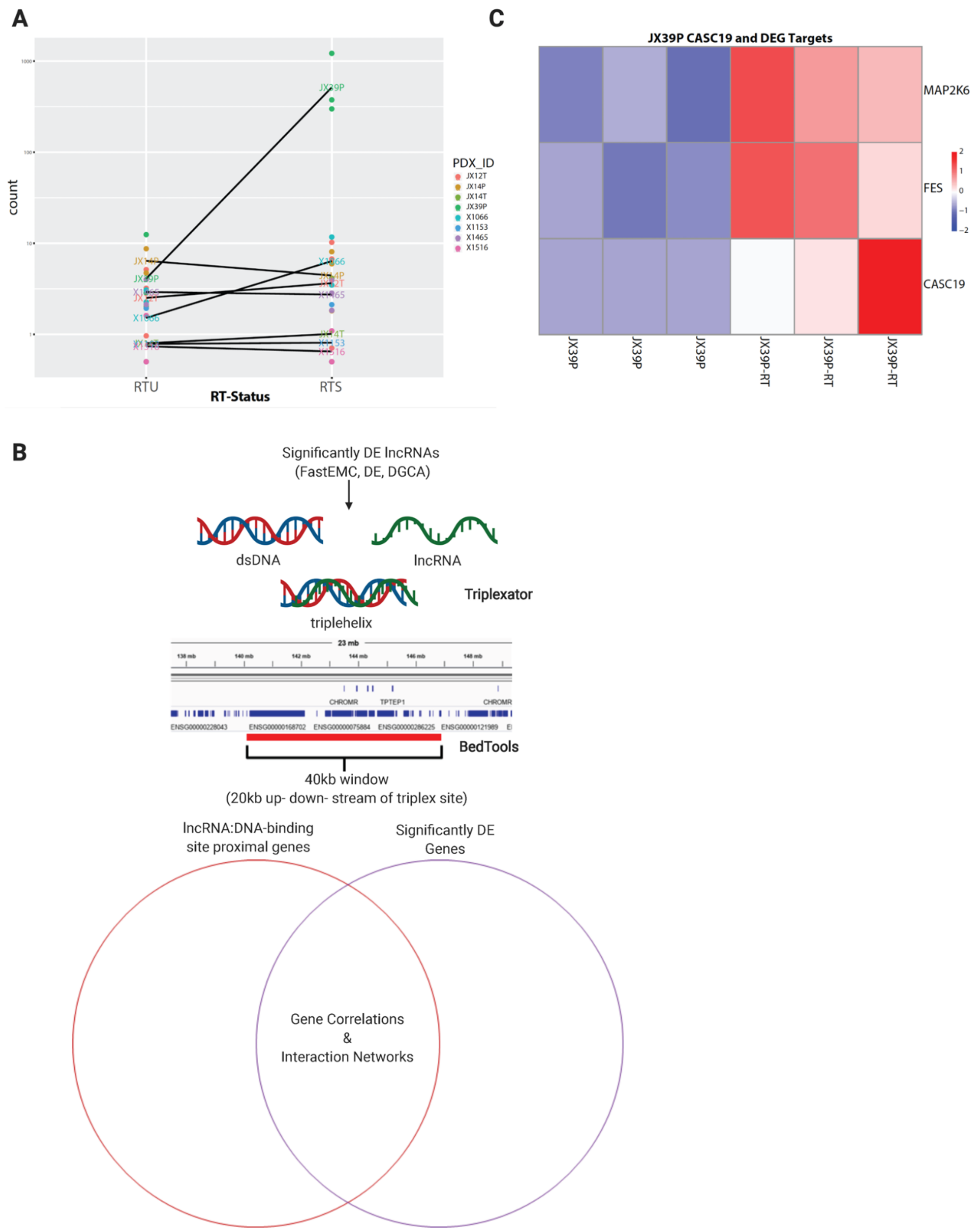

Supplementary Figure S2: lncRNA Examination. A, CASC19 transcript expression (log10 scaled) in RTU (sensitive) versus RTS (resistant) pairs. B, z-score of expression of CASC19 along with associated DEGs in the JX39P pair. C, Workflow for determining potential cis-regulatory action of lncRNAs. lncRNAs of interest are identified in pairwise analysis from machine learning, differential expression, and differential correlation analysis. The sequences of these lncRNAs are compared to a double stranded DNA library to predict triple helix binding sites using the program Triplexator. Then bedtools is used to look 20kb up and down stream of

each triplex site to identify proximal genes to the lncRNA:DNA binding sites. The proximal genes list are then compared to the pairwise differential expression results to find genes common to both sets. These genes are purported cis-regulatory targets of the significant lncRNAs. Created with BioRender.

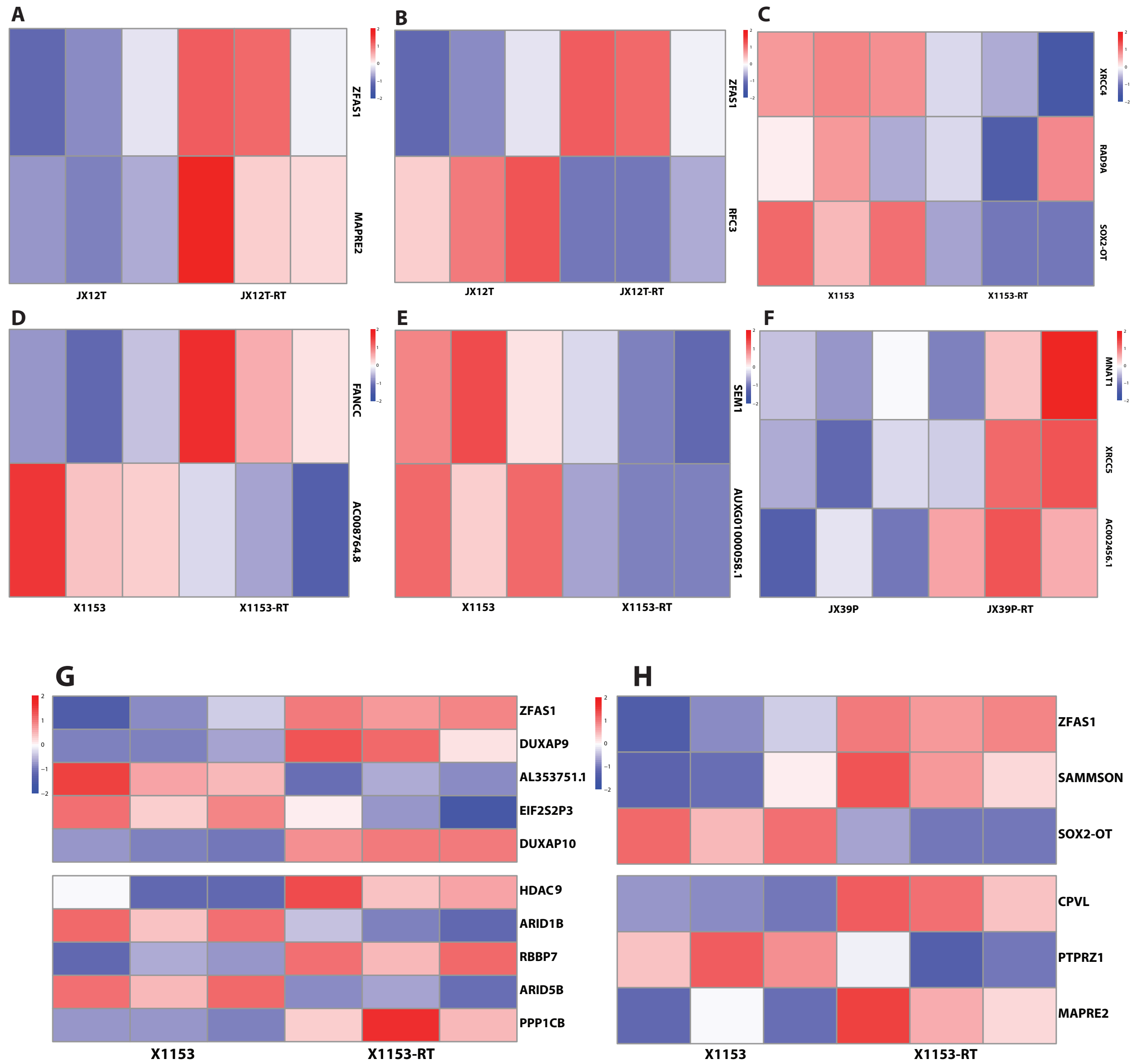

Supplementary Figure S3: lncRNA correlations with lncRNA:DNA binding site significantly differentially expressed proximal genes. (A) Positive correlation of expression of ZFAS1 with stemness related gene MAPRE2. (B) Negative correlation of expression of ZFAS1 with cell cycle related gene RFC3. (C) Correlations of expression of SOX2-OT with non-homologous end joining DNA repair genes XRCC4 and RAD9A. (D) Negative correlation of AC008764.8 with Fanconi anemia-related gene FANCC. (E) Positive correlation of AUXG01000058.1 with homologous recombination gene SEM1. (F) Correlations of expression of AC002456.1 with DNA damage response genes XRCC5 and MNAT1. (G) Patterns of expression of multiple

lncRNAs with multiple chromatin remodeling genes in X1153. (H) Patterns of expression of multiple lncRNAs with multiple stemness related genes.

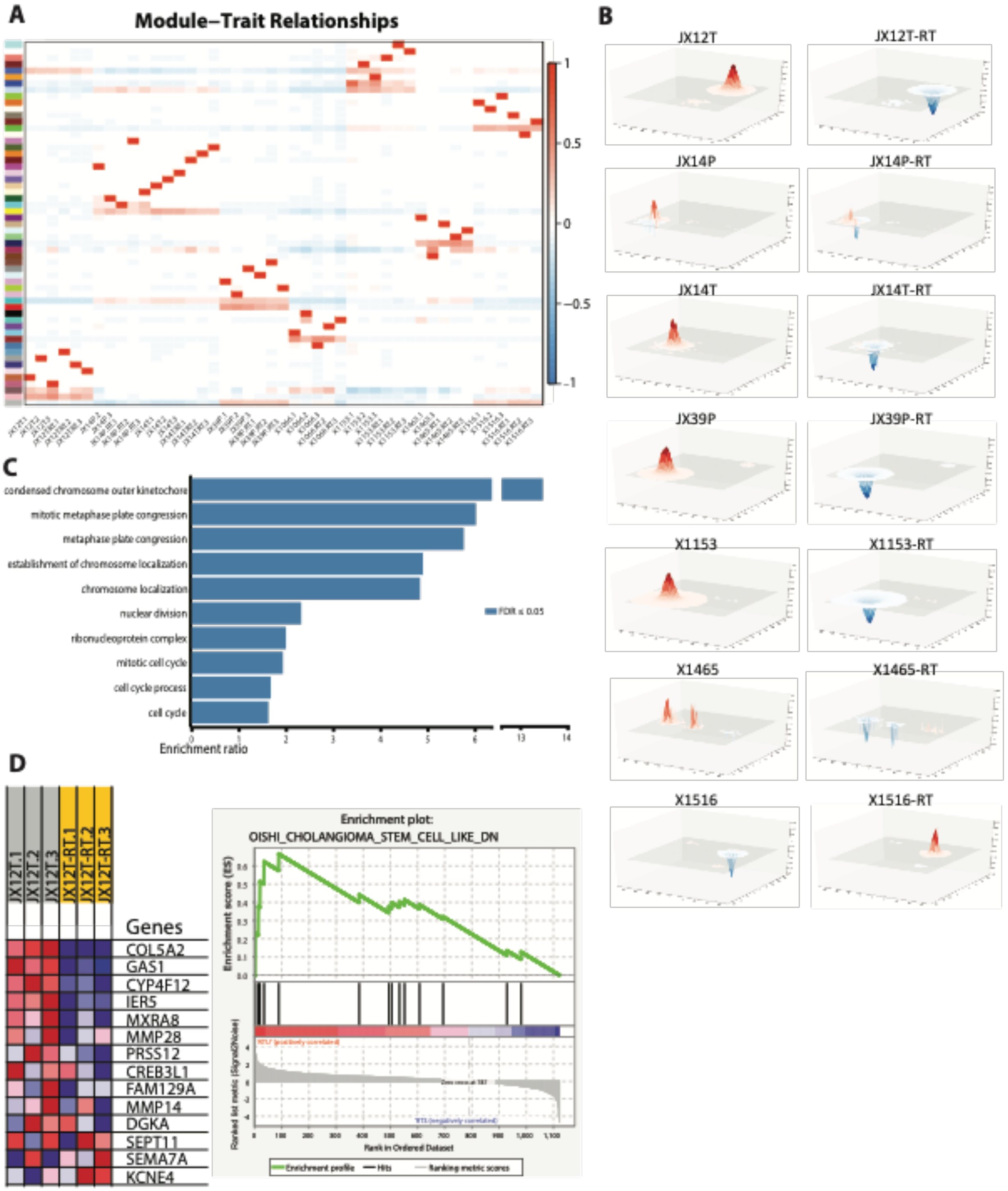

Supplementary Figure S4: Distinct gene modules define PDX by patient of origin while differential regulation within modules define RTU verses RTS. A, gene module Pearson correlations across PDX samples. Colored boxes along y-axis indicate gene modules. B, Gene

Terrain maps of gene module regulation in RTS and RTU averaged across replicates (x,y- gene module coordinates; z- module expression fold-change). C, Over representation analysis significantly enriched terms of genes within the JX12T modules. False discovery rate (FDR) was calculated for gene set enrichment. D, enrichment of genes downregulated in stem cell-like cholangiocarcinoma (Oishi) in JX12T-related modules.

A JX14P-RT

MSK1 (RPS6KA5)	O75582
CAMK4	Q16566
Pim3 (AL549548)	P58750
CHK2 (CHEK2)	O96017
PIM1	P11309
HCK	P08631
PKCe (PRKCE)	Q02156
PKCd (PRKCD)	Q05655
PIM2	Q9P1W9
PKCa (PRKCA)	P17252
BRK	Q13882
PKCh (PRKCH)	P24723
LYN	P07948
PKCg (PRKCG)	P05129
PYK2 (PTK2B)	Q14289
PRKX	P51817
SGK	O00141
YES1	P07947
SRC	P12931
BLK	P51451

B JX14T-RT

Pim3 (AL549548)	P58750
PIM1	P11309
BRK	Q13882
ZAP70	P43403
ERBB3	P21860
BMX	P51813
ARG (ABL2)	P42684
ABL	P00519
PIM2	Q9P1W9
JNK1 (MAPK8)	P45983
JNK3 (MAPK10)	P53779
MSK1 (RPS6KA5)	O75582
SYK	P43405
EGFR	P00533
CDK3	Q00526
CDK1 (CDC2)	P06493
HCK	P08631
JNK2 (MAPK9)	P45984
p70S6Kb (RPS6KB2)	Q9UBS0
PYK2 (PTK2B)	Q14289

C JX39P-RT

BRK	Q13882
HCK	P08631
PYK2 (PTK2B)	Q14289
LYN	P07948
SRC	P12931
YES1	P07947
ERBB3	P21860
CTK (MATK)	P42679
BLK	P51451
FYN	P06241
Pim3 (AL549548)	P58750
PIM1	P11309
PIM2	Q9P1W9
ABL	P00519
ARG (ABL2)	P42684
MSK1 (RPS6KA5)	O75582
PKG1 (PRKG1)	Q13976
PKG2 (PRKG2)	Q13237
JNK1 (MAPK8)	P45983
JNK3 (MAPK10)	P53779

D X1465-RT (Increased)

mTOR/FRAP	P42345
ERK5 (MAPK7)	Q13164
JNK2 (MAPK9)	P45984
PITSLRE (CDC2L1)	Q9UQ88
CDK7	P50613
IKKE	Q14164
JNK1 (MAPK8)	P45983
JNK3 (MAPK10)	P53779
PSKH1 (HUMPSKB)	P11801
IKKb (IKBKINASE)	O14920

X1465-RT (Decreased)

Pim3 (AL549548)	P58750
PIM1	P11309
PIM2	Q9P1W9
ATR	Q13535
PKG1 (PRKG1)	Q13976
p70S6Kb (RPS6KB2)	Q9UBS0
PKG2 (PRKG2)	Q13237
p70S6K (RPS6KB1)	P23443
RSK3 (RPS6KA1)	Q15349
RSK1 (RPS6KA2)	Q15418
RSK4 (RPS6KA6)	Q9UK32
MEK1 (MAP2K1)	Q02750
DNAPK/PRKDC	P78527
MEK2 (MAP2K2)	P36507
MSK1 (RPS6KA5)	O75582
PKACa (PRKACA)	P17612
PKACb PRKACB	P22694
PRKX	P51817
AKT2	P31751
AKT1	P31749

Supplementary Figure S5: Upstream kinase scores for R&D Systems Proteome Profiler PhosphoKinase Array. Upstream kinase scoring from Kinexus Phosphonet was manually entered

for each available residue of >25% (mean of 2) altered array spots (See Figure 6). Kinases were pulled from available Kinexus Phosphonet database, and a sum of the top 10 kinases Phosphonet scores were compiled per each kinase and ranked. The top 20 kinases are shown, with overlapping kinases from PamStation kinomics screen mean final score >2.0 are highlighted in green. A residue correction factor (i.e., multilply Kinexus score by 1, 0.5, 0.25, depending on number of residues per phospho-target on the array) was applied. A, JX14P-RT; B, JX14T-RT; C, JX39P-RT; D, X1465-RT. Created with BioRender.

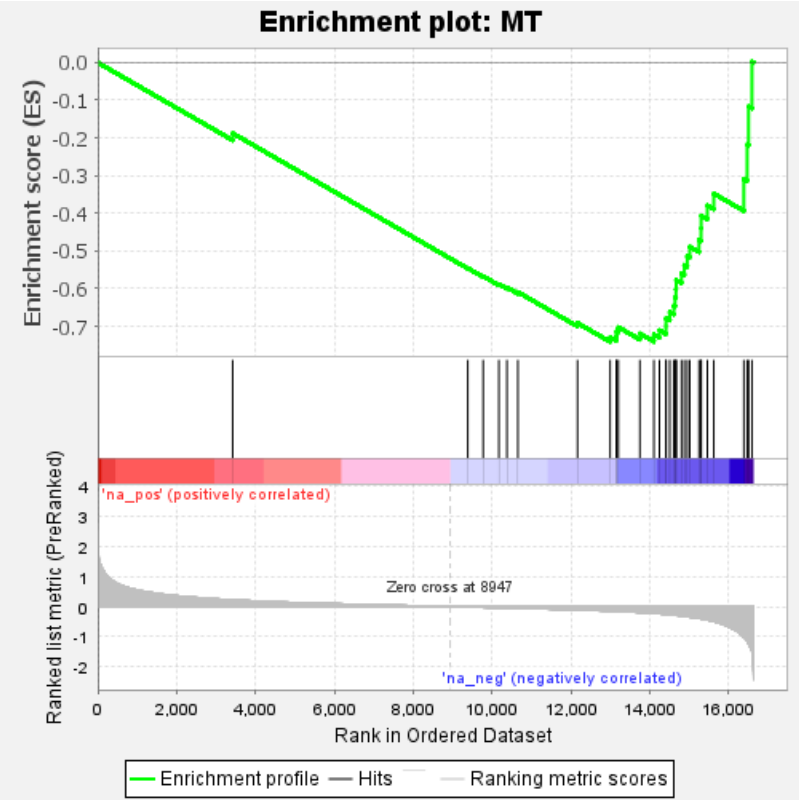

Supplementary Figure S6: Gene set enrichment analysis for mouse-specific transcriptome. For the positional gene sets in the mouse-specific transcriptome, only the mitochondrial genes were significantly altered with mitochondrial genes having decreased expression in the mouse cells that infiltrate tumors that have been radiation-selected. The GSEA plot is shown.

Descriptions of Supplementary Files:

Supplementary File S1: Pearson correlations of lncRNA expression with lncRNA:DNA binding site proximal genes. Top positive and negative correlations for lncRNA:proximal gene pairs. Genes highlighted yellow are directly related to cancer pathways. Green highlighted genes indicate genes common to both DGCA and FastEMC/DEG analysis. Blue highlighted gene is a cancer-related fusion product of interest.

Supplementary File S2: WIPER gene interaction networks using lncRNAs and lncRNA:DNA binding site proximal genes. Diamonds indicate lncRNAs. Circles indicate proximal genes.

Supplementary File S3: Pairwise upstream kinase predictions. Kinases ranked by mean final kinase score. Mean kinase statistic indicates direction of kinase activity enrichment (red positive enrichment in RTS, blue positive enrichment in RTU.)

Supplementary File S4: GeneGo pathway analysis based on pairwise differentially expressed lncRNA:DNA proximal genes alone (DEG (40kb)) or based on transcriptomics integrated with pairwise kinomics (DEG + Kinomics). Shaped are standard GeneGo Symbols. Smaller circle offset in upper-right corner color indicates enrichment in RTS (red) or in RTU (blue). Arrows indicate a neutral relationship (grey), positive relationship (green), or negative relationship (red).

References:

1. Patro, R., Duggal, G., Love, M.I., et al. 2017. Salmon: fast and bias-aware quantification of transcript expression using dual-phase inference. *Nat Methods* 14:417-419.
2. Zheng, H., Brennan, K., Hernaez, M., et al. 2019. Benchmark of long non-coding RNA quantification for RNA sequencing of cancer samples. In *Gigascience*.
3. Stackhouse, C.T., Rowland, J.R., Shevin, R.S., et al. 2019. A Novel Assay for Profiling GBM Cancer Model Heterogeneity and Drug Screening. *Cells* 8.
4. Love, M.I., Huber, W., and Anders, S. 2014. Moderated estimation of fold change and dispersion for RNA-seq data with DESeq2. *Genome Biology* 15.
5. McKenzie, A.T., Katsyv, I., Song, W.M., et al. 2016. DGCA: A comprehensive R package for Differential Gene Correlation Analysis. *BMC Syst Biol* 10:106.
6. Buske, F.A., Bauer, D.C., Mattick, J.S., et al. 2012. Triplexator: detecting nucleic acid triple helices in genomic and transcriptomic data. *Genome Res* 22:1372-1381.
7. Antonov, I., Marakhonov, A., Zamkova, M., et al. 2018. ASSA: Fast identification of statistically significant interactions between long RNAs. *J Bioinform Comput Biol* 16:1840001.
8. Subramanian, A., Tamayo, P., Mootha, V.K., et al. 2005. Gene set enrichment analysis: a knowledge-based approach for interpreting genome-wide expression profiles. *Proc Natl Acad Sci U S A* 102:15545-15550.
9. Yue, Z., Willey, C.D., Hjelmeland, A.B., et al. 2019. BEERE: a web server for biomedical entity expansion, ranking and explorations. *Nucleic Acids Res* 47:W578-W586.
10. Qian You, S.F., Jake Yue Chen. 2010. Gene Terrain: Visual Exploration of Differential Gene Expression Profiles Organized in Native Biomolecular Interaction Networks. *Information Vizualization* 9:1-12.
11. Zerbino, D.R., Wilder, S.P., Johnson, N., et al. 2015. The ensembl regulatory build. *Genome Biol* 16:56.
12. Yue, Z., Kshirsagar, M.M., Nguyen, T., et al. 2015. PAGER: constructing PAGs and new PAG–PAG relationships for network biology. In *Bioinformatics*. i250-257.
13. Zongliang Yue, T.N., Eric Zhang, Jianyi Zhang, Jake Y. Chen. 2019. WIPER: Weighted in-Path Edge Ranking for biomolecular association networks. *Quantitative Biology* 7:331-326.
14. Gambella, A., Senetta, R., Collemi, G., et al. 2020. NTRK Fusions in Central Nervous System Tumors: A Rare, but Worthy Target. *Int J Mol Sci* 21.

15. Zhang, B., Kirov, S., and Snoddy, J. 2005. WebGestalt: an integrated system for exploring gene sets in various biological contexts. *Nucleic Acids Res* 33:W741-748.
16. Langfelder, P., and Horvath, S. 2008. WGCNA: an R package for weighted correlation network analysis. *BMC Bioinformatics* 9:559.
