# Supplementary figures and images for: An in vivo model of glioblastoma radiation resistance identifies long noncoding RNAs and targetable kinases
Source: JCI Insight. 2022 Aug 22;7(16):e148717. doi: 10.1172/jci.insight.148717 (PMC9462495; doi:10.1172/jci.insight.148717)

Global

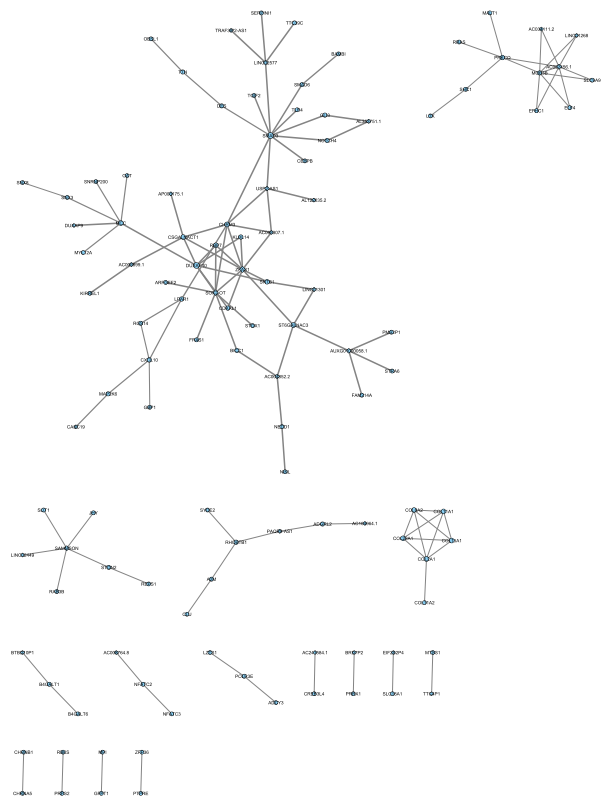

JX12T

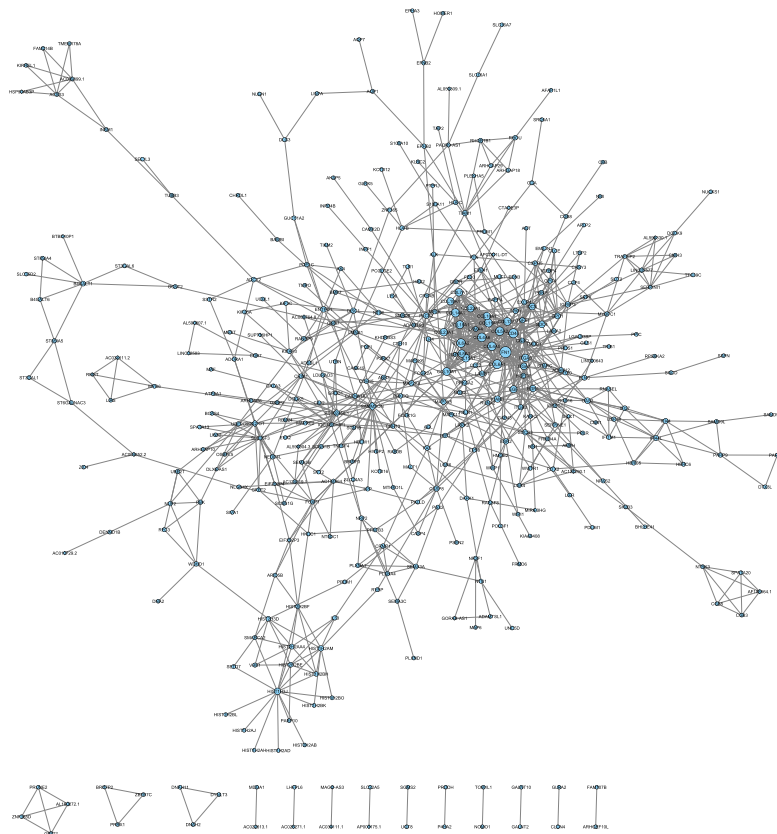

JX14P

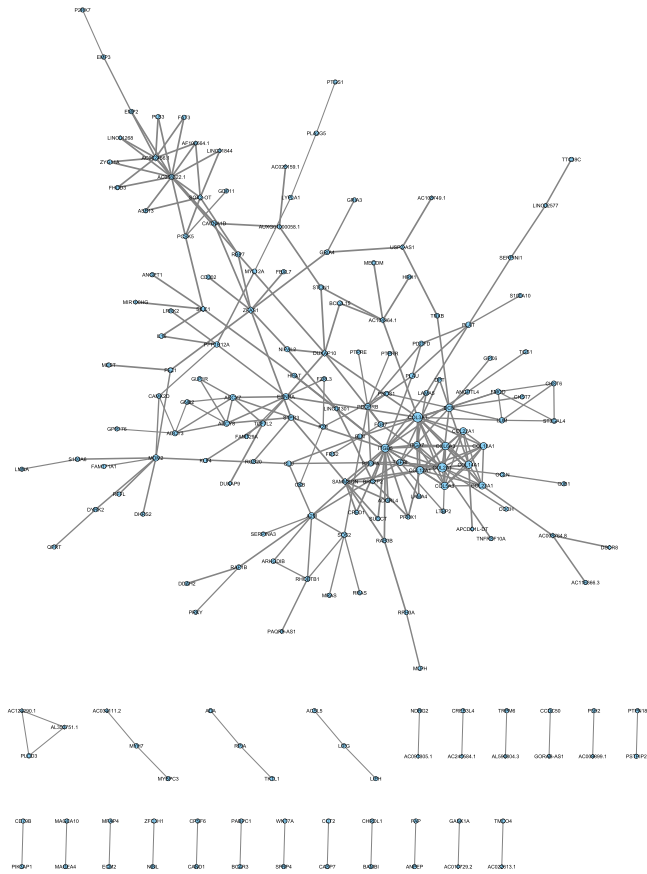

JX14T

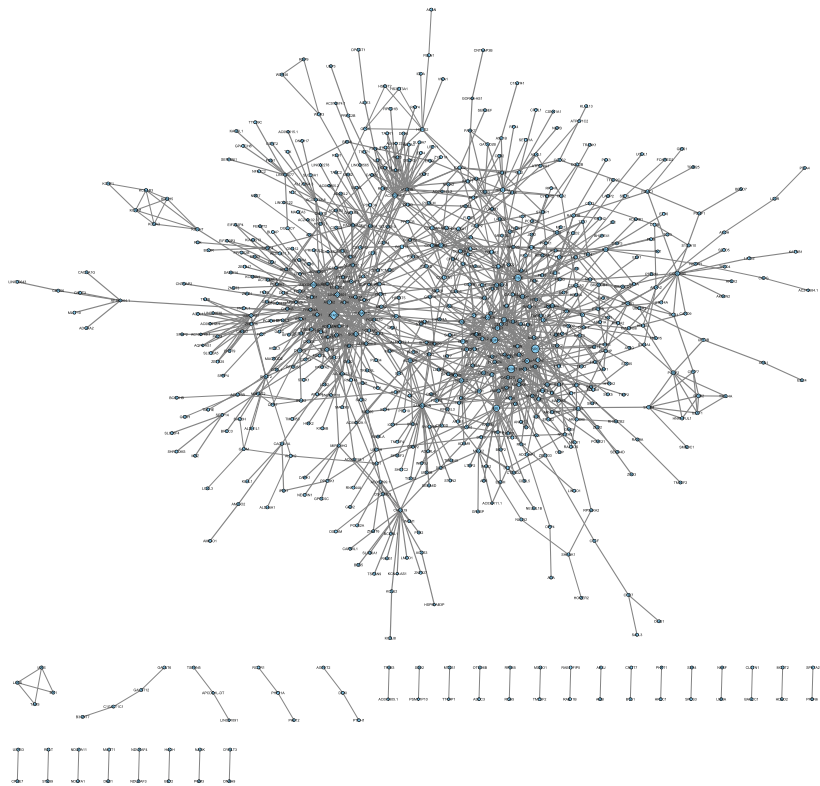

JX39P

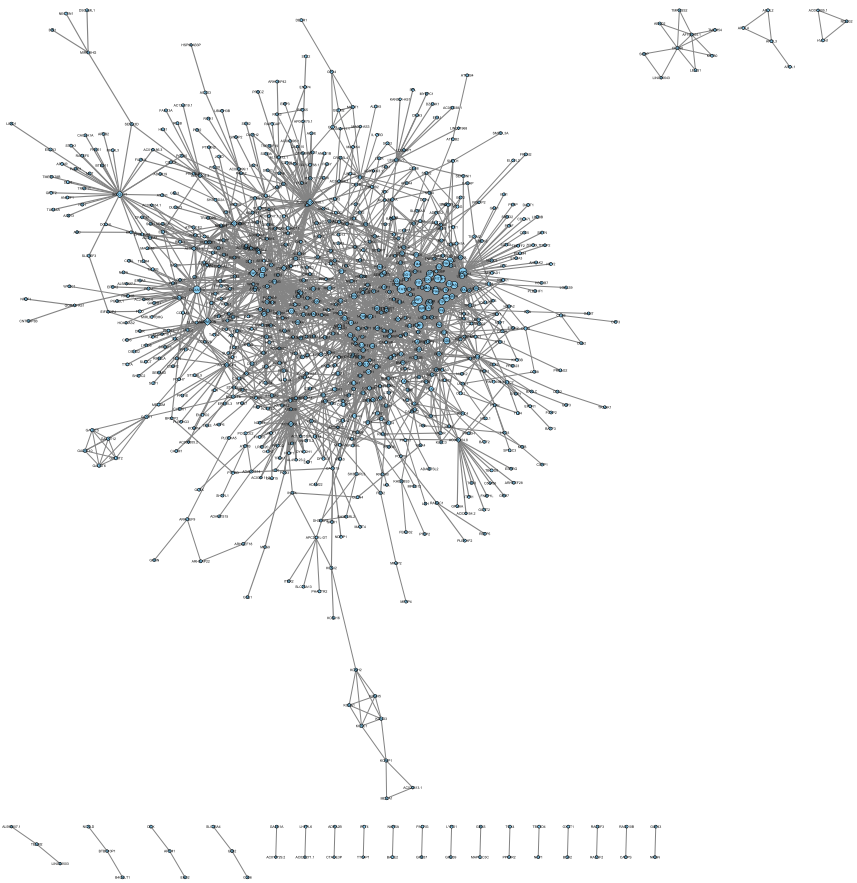

X1066

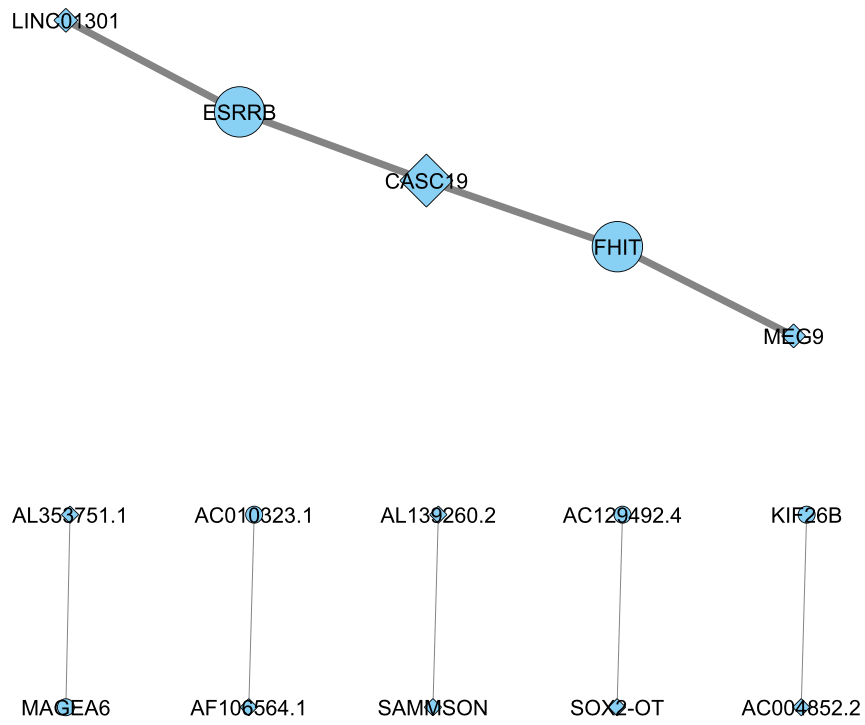

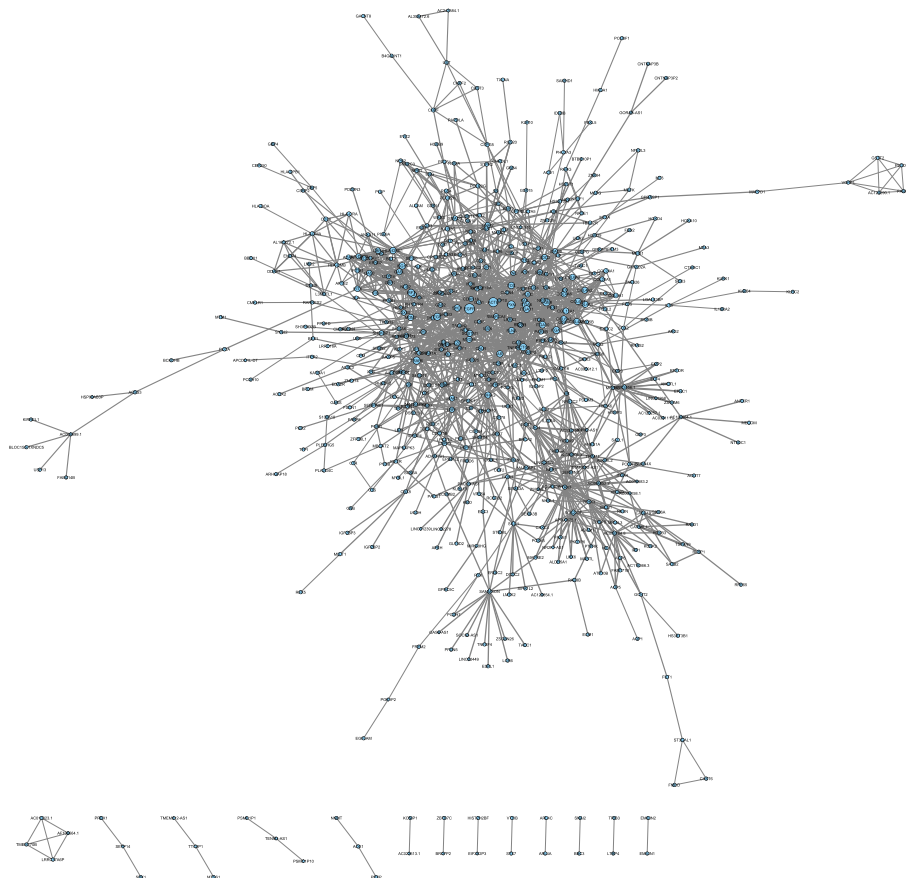

X1516

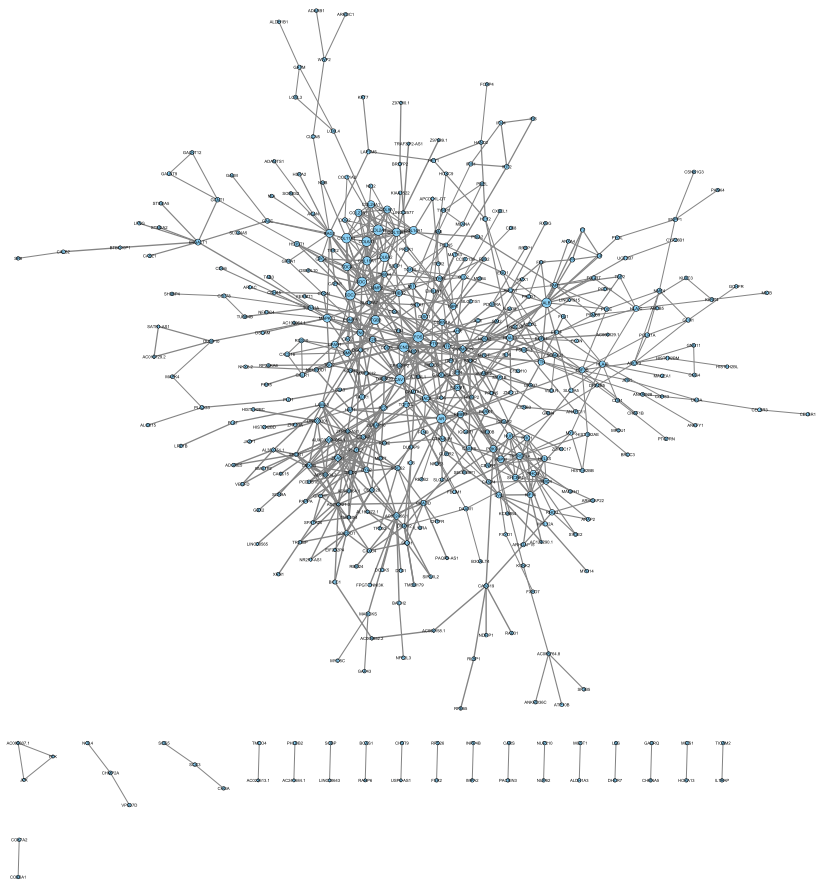

Supplement: Supplemental data set 2 [file jciinsight-7-148717-s019.pdf]
