# Supplementary material for: An in vivo model of glioblastoma radiation resistance identifies long noncoding RNAs and targetable kinases
Source: JCI Insight. 2022 Aug 22;7(16):e148717. doi: 10.1172/jci.insight.148717 (PMC9462495; doi:10.1172/jci.insight.148717)

JX12T DEG (40kb)

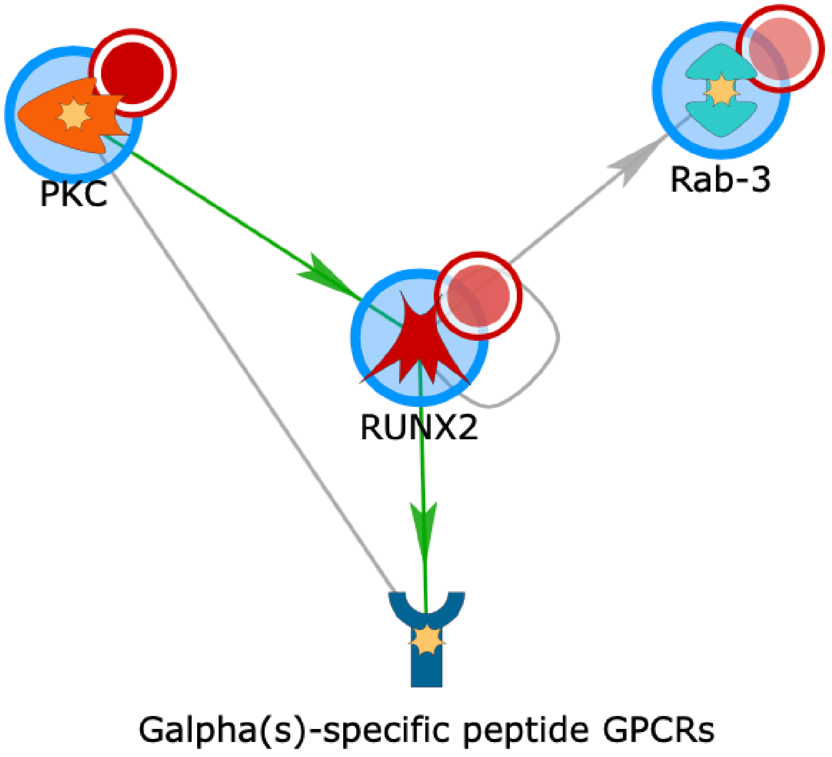

JX12T DEG + Kinomics

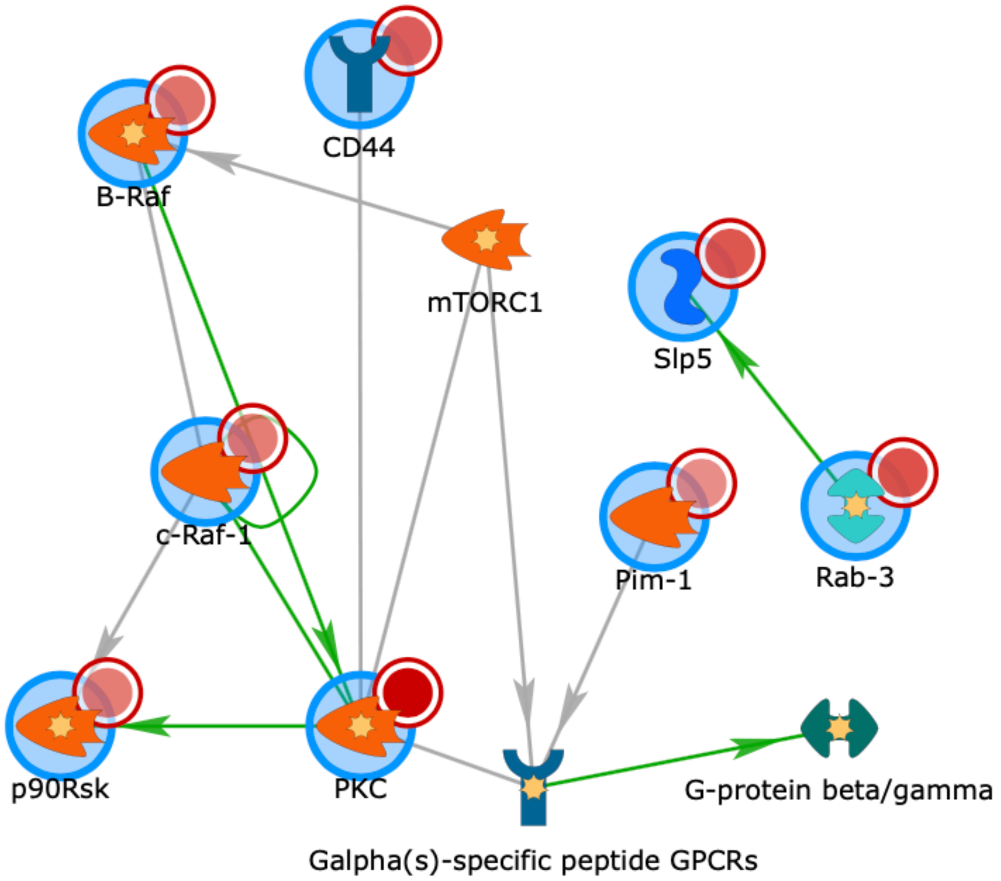

JX14P DEG (40kb)

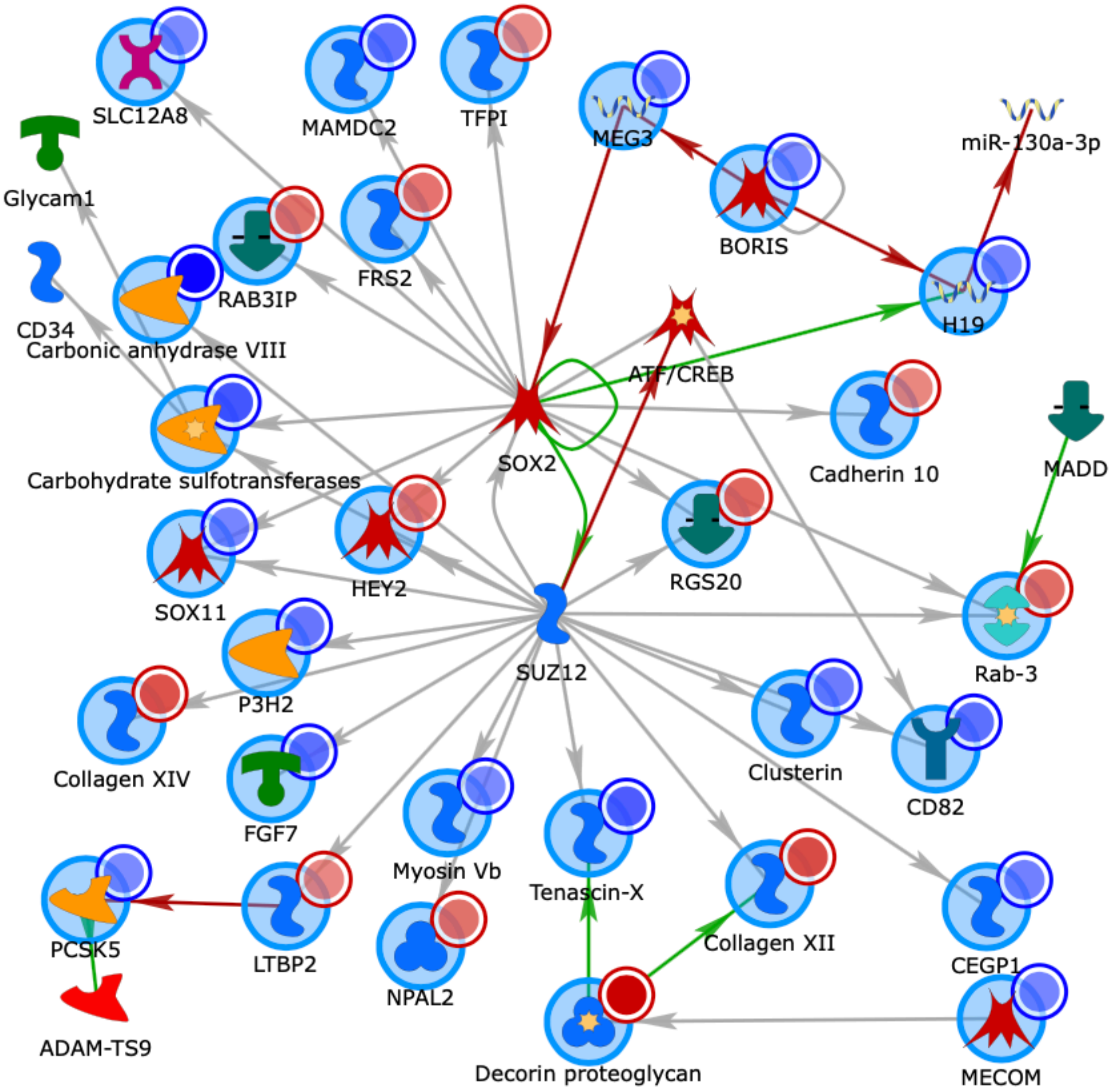

JX14P DEG + Kinomics

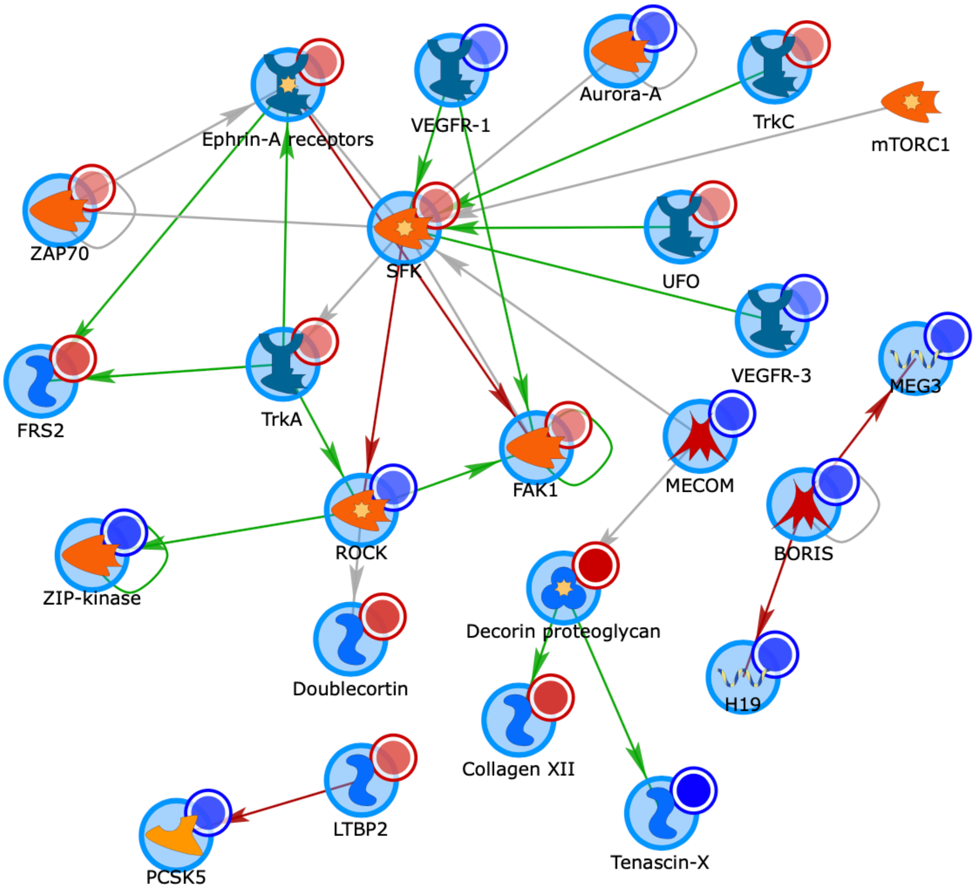

JX14T DEG (40kb)

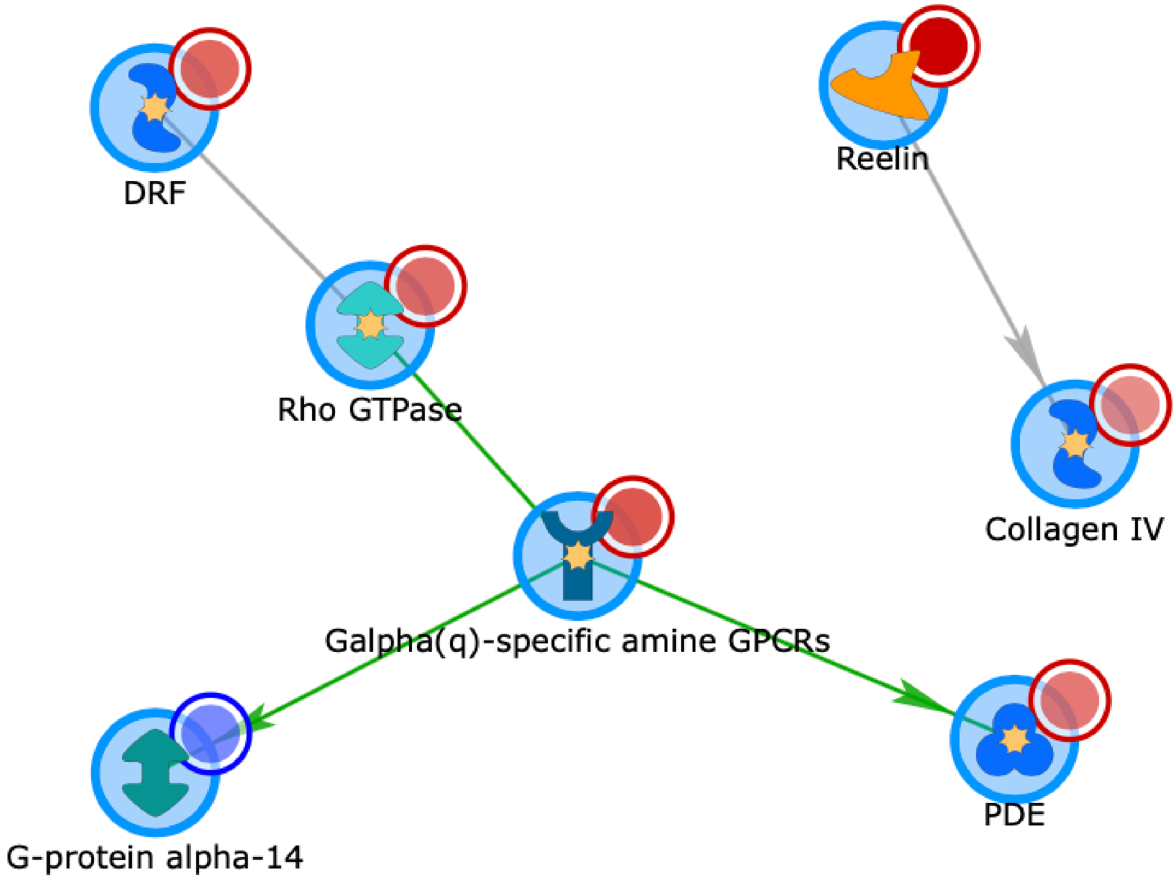

JX14T DEG + Kinomics

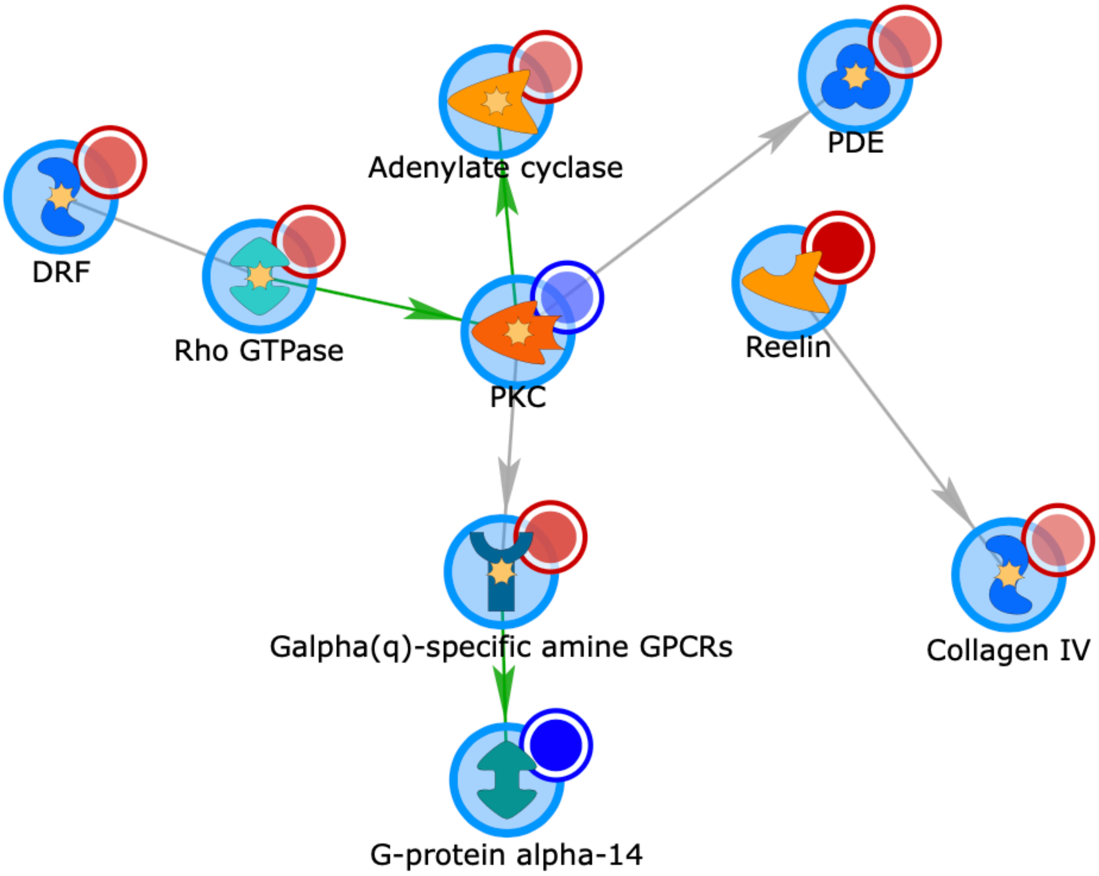

JX39P DEG (40kb)

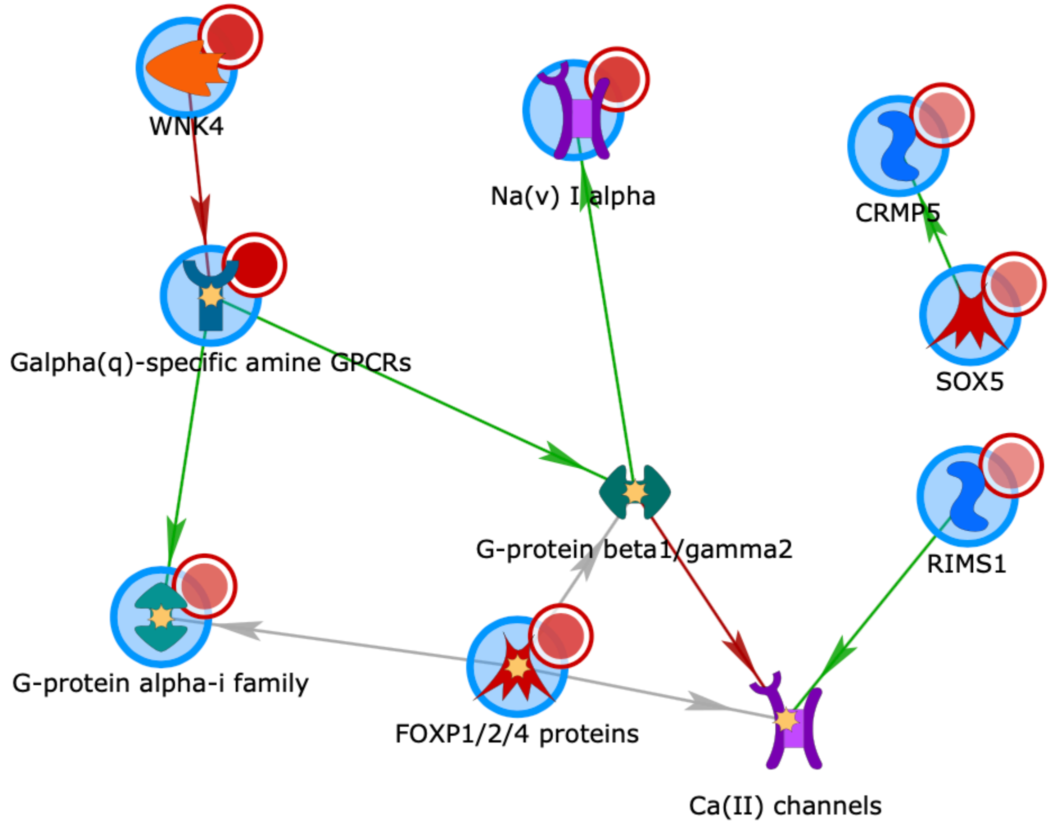

JX39P DEG + Kinomics

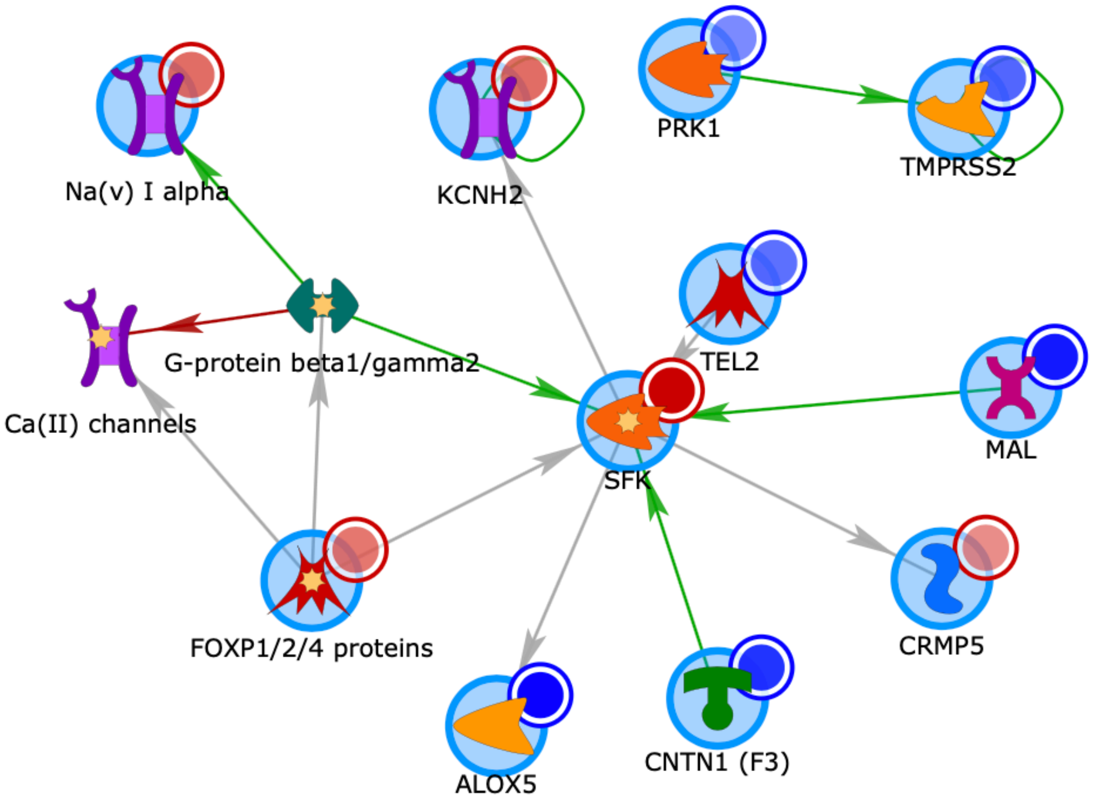

X1153 DEG (40kb)

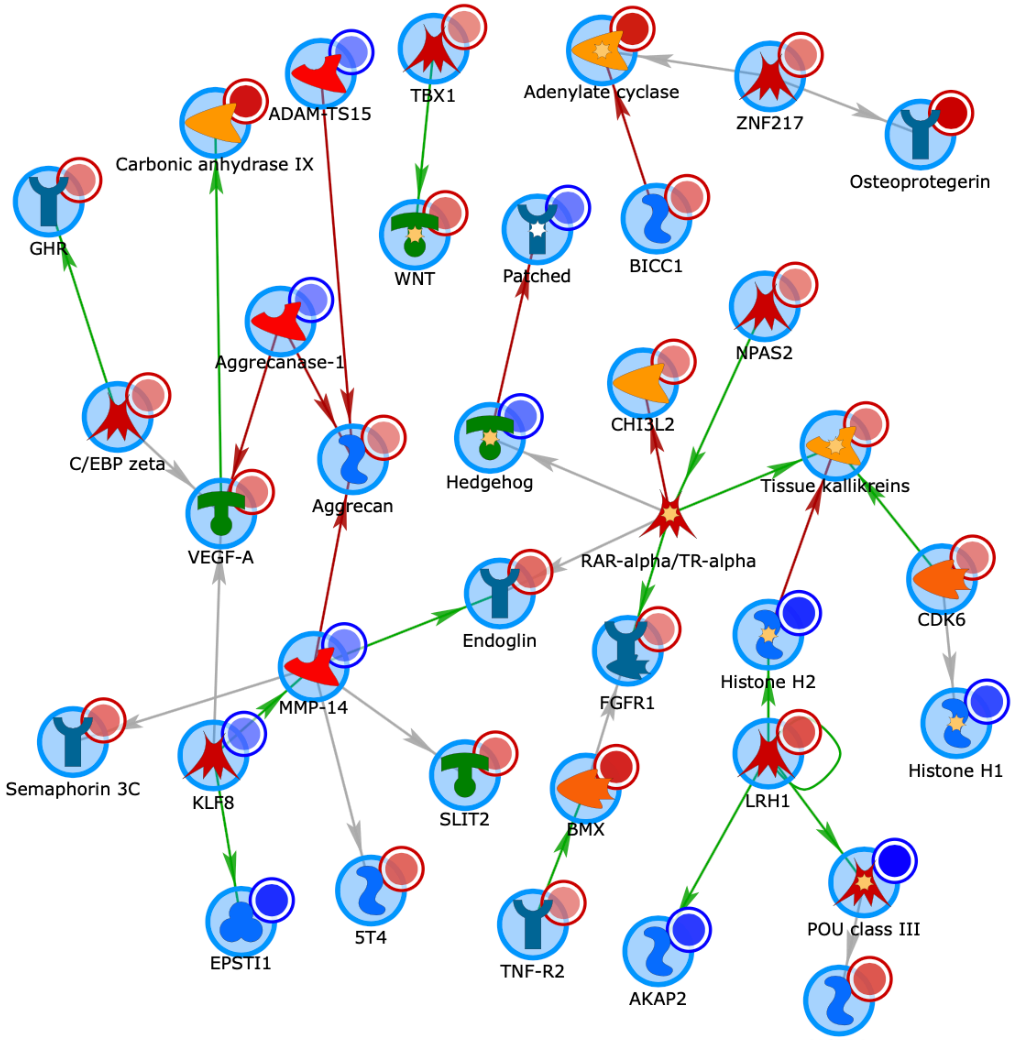

X1153 DEG + Kinomics

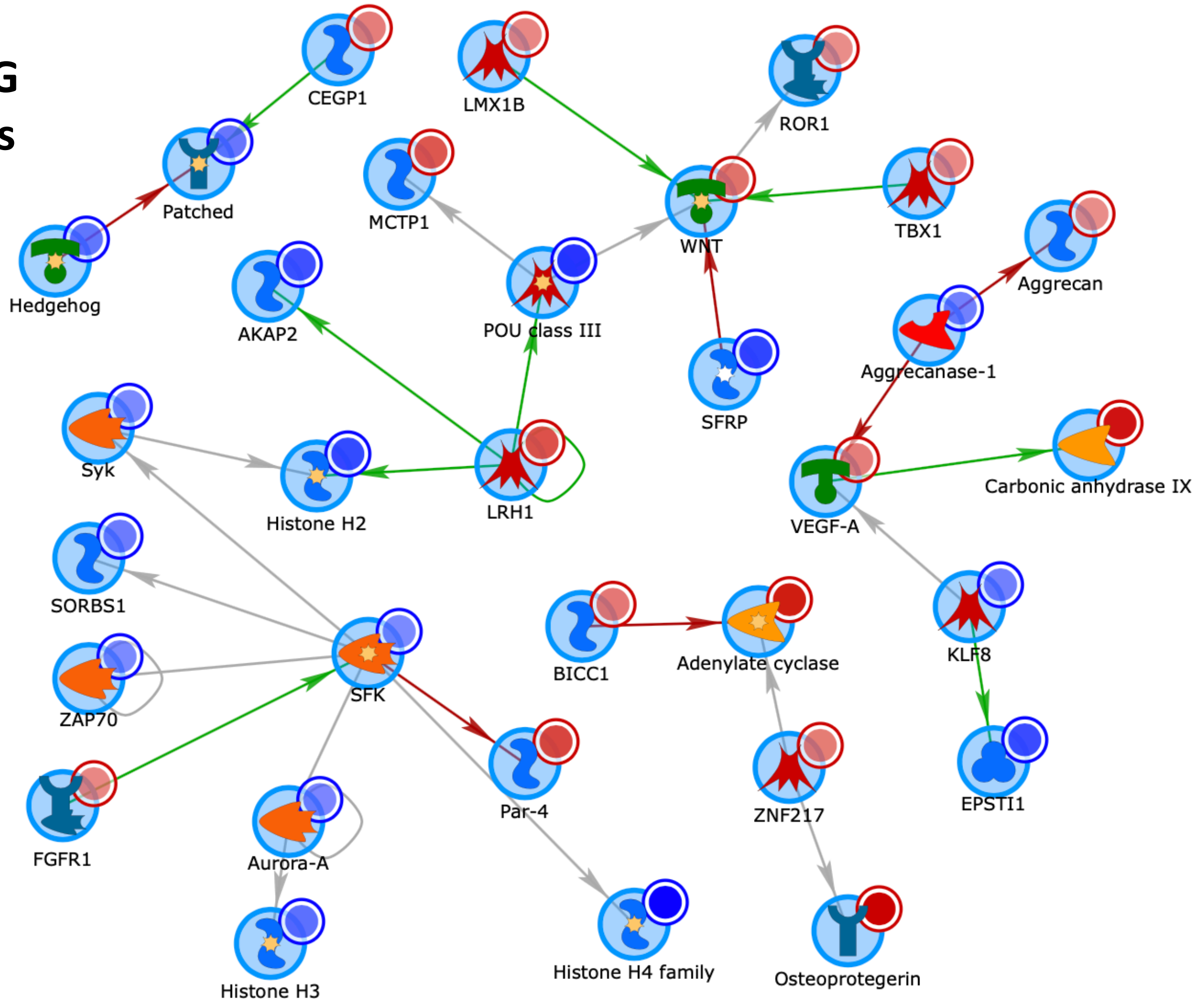

X1465 DEG (40kb)

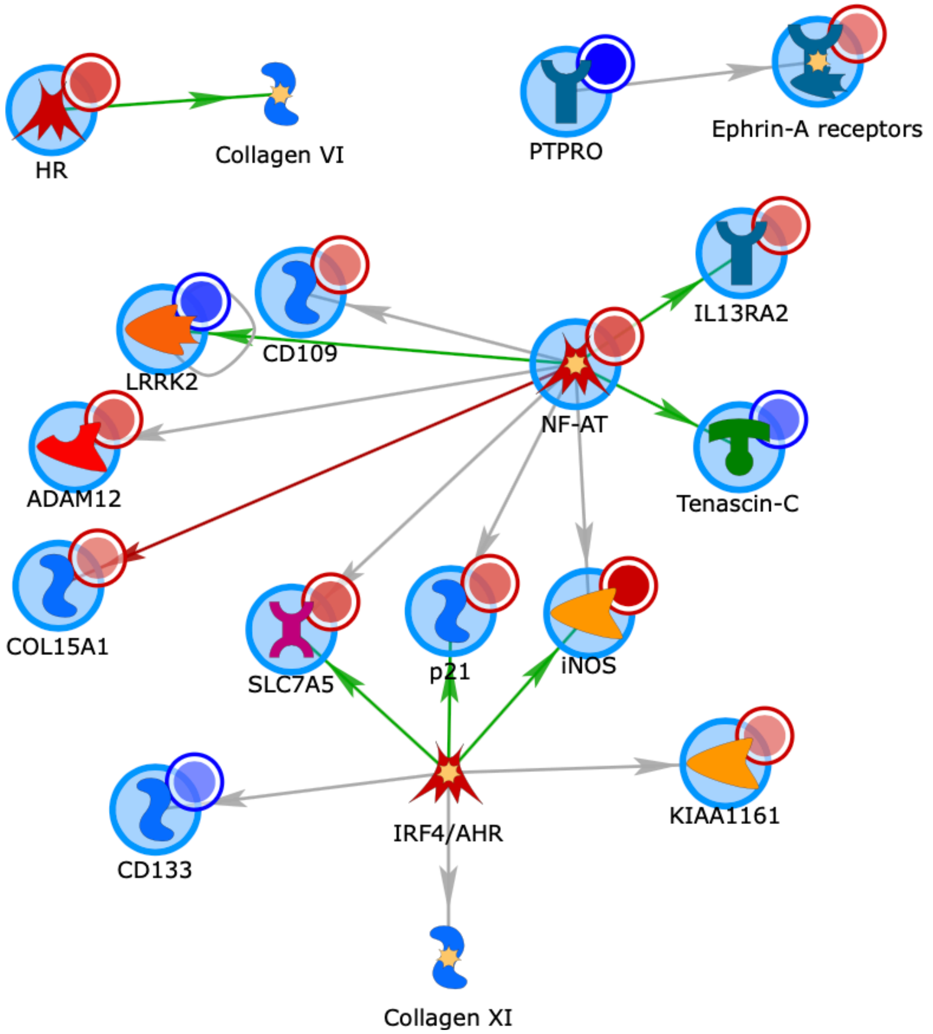

X1465 DEG + Kinomics

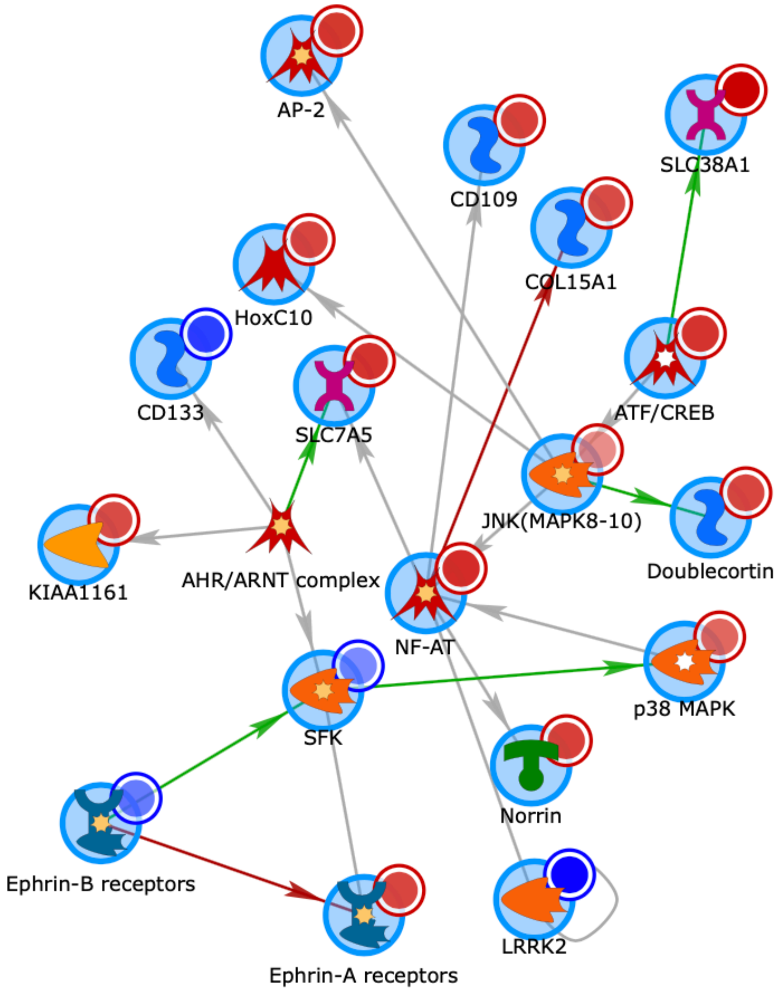

X1516 DEG (40kb)

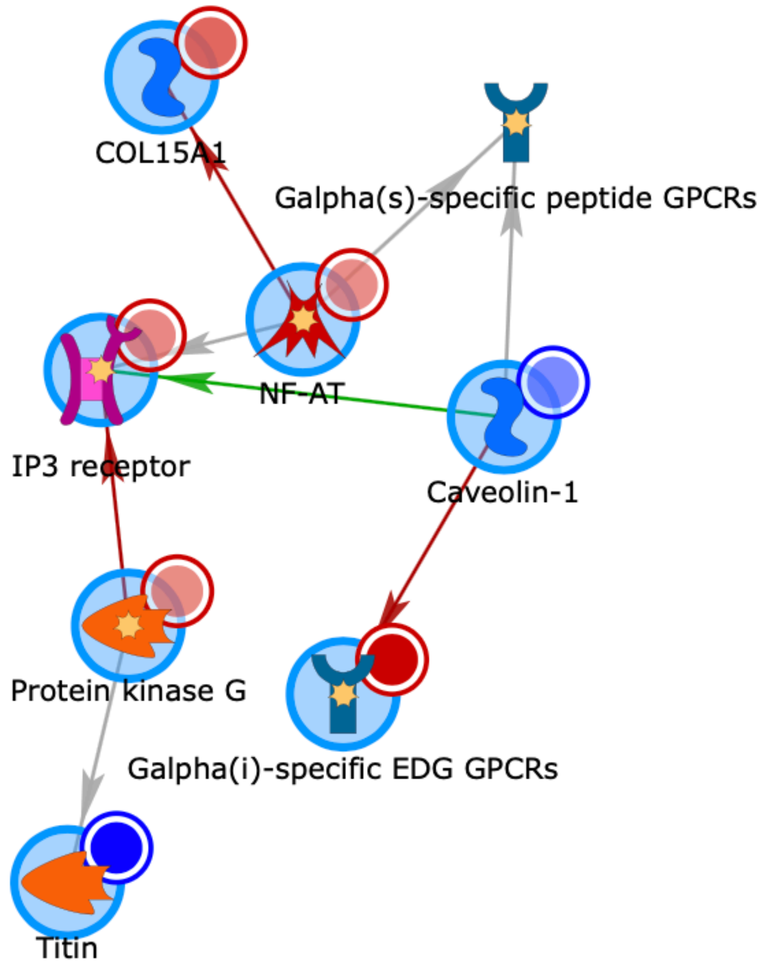

X1516 DEG + Kinomics

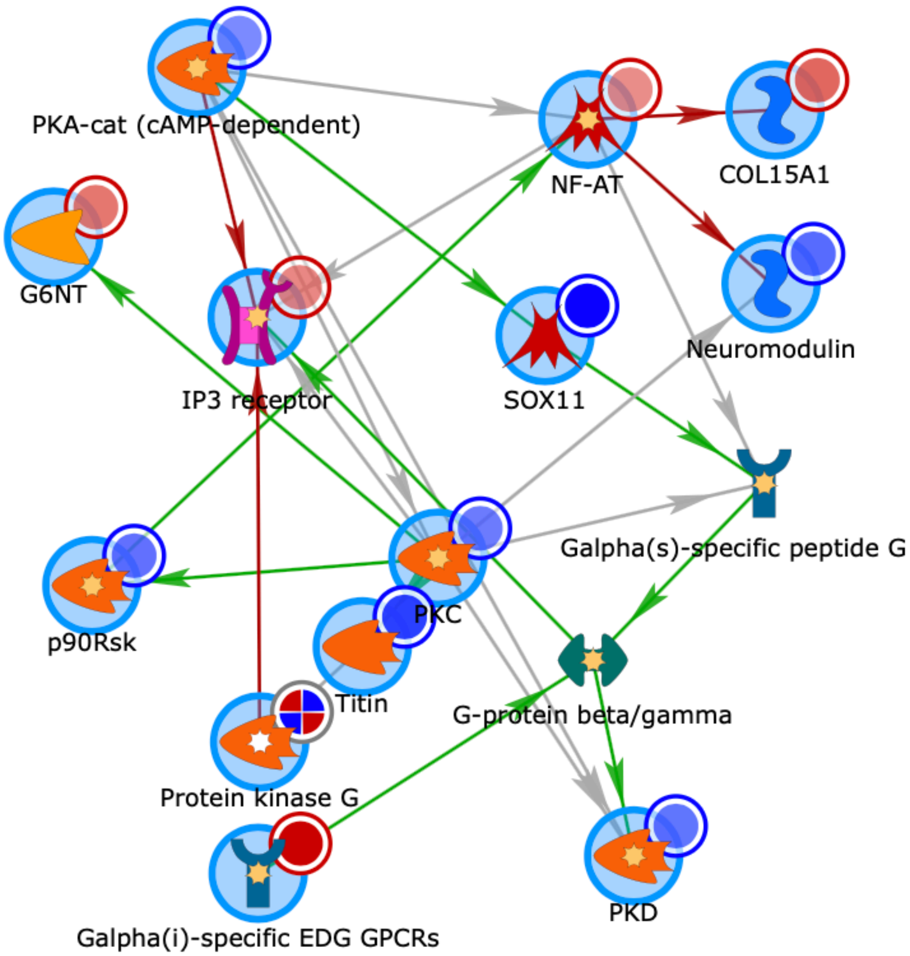

Supplement: Supplemental data set 4 [file jciinsight-7-148717-s021.pdf]
